# A novel proton transfer mechanism in the SLC11 family of divalent metal ion transporters

**DOI:** 10.1038/s41598-017-06446-y

**Published:** 2017-07-28

**Authors:** Jonai Pujol-Giménez, Matthias A. Hediger, Gergely Gyimesi

**Affiliations:** Institute of Biochemistry and Molecular Medicine and National Center of Competence in Research, NCCR TransCure, University of Bern, Bern, Switzerland

## Abstract

In humans, the H^+^-coupled Fe^2+^ transporter DMT1 (SLC11A2) is essential for proper maintenance of iron homeostasis. While X-ray diffraction has recently unveiled the structure of the bacterial homologue ScaDMT as a LeuT-fold transporter, the exact mechanism of H^+^-cotransport has remained elusive. Here, we used a combination of molecular dynamics simulations, *in silico* p*K*_*a*_ calculations and site-directed mutagenesis, followed by rigorous functional analysis, to discover two previously uncharacterized functionally relevant residues in hDMT1 that contribute to H^+^-coupling. E193 plays a central role in proton binding, thereby affecting transport properties and electrogenicity, while N472 likely coordinates the metal ion, securing an optimally “closed” state of the protein. Our molecular dynamics simulations provide insight into how H^+^-translocation through E193 is allosterically linked to intracellular gating, establishing a novel transport mechanism distinct from that of other H^+^-coupled transporters.

## Introduction

## Biology and Relevance

Iron is a key element in the metabolism of almost all living organisms, being involved in the transport and storage of oxygen and the catalysis of redox reactions in humans. In human intestine, uptake of ferrous ion (Fe^2+^) from the diet is mediated primarily by the divalent metal ion transporter 1 (DMT1, also SLC11A2)^1^, expressed in the brush border membrane of enterocytes in the duodenum^2, 3^. Mutations in the SLC11A2 gene can lead to microcytic anemia^3–6^. The availability of DMT1 for oral medication on the luminal side of the intestine makes it an interesting drug target for iron overload disorders.

The first mammalian divalent metal ion transporter (DMT1/SLC11A2) was identified from rat and mouse^4, 7^, and was shown to couple the uptake of several transition metal ions (Fe^2+^, Mn^2+^ and Cd^2+^) to the cotransport of H^+^^7, 8^. Interestingly, the stoichiometry of the transported H^+^:Fe^2+^ can either be high (~10:1) at low pH, causing a large H^+^ flux that is uncoupled from Fe^2+^ uptake^9^ or transport can be H^+^-independent at high pH^10^. Additionally, the transporter is able to mediate a H^+^-leak current in absence of substrate^10^. Analysis of two conserved histidine residues (H267 and H272) in the rat DMT1 transporter pinpointed H272 to be responsible for coupling Fe^2+^ and H^+^ transport^10^, but structural evidence suggests that this residue is inaccessible from the extracellular medium^11–13^. In contrast, while the rat H267A mutant had functional characteristics similar to wild-type, the corresponding mutation in a prokaryotic homologue was found to cause H^+^-independent metal ion uptake^13^. Thus, the exact mechanism of coupling and the role of either histidine residue in proton-coupled transport still remain elusive.

Structurally, SLC11 transporters belong to the “Amino acid-Polyamine-organoCation” (APC) superfamily^11–13^, containing Na^+^- and H^+^-coupled secondary transporters and symporters, some of which are particularly well characterized, such as LeuT^14–17^. In the structure of the H^+^-coupled transporter ApcT, K158, a residue with a basic side-chain located on TMH 5 was shown to be responsible for proton cotransport, extending into the binding pocket harboring the Na2 sodium ion in Na^+^-coupled family members^17, 18^. In CaiT, which is a H^+^-independent transporter of the same fold family, R262 occupies the location analogous to Na2 in LeuT^19^. Due to the high estimated p*K*_*a*_ of R262, it was proposed that it does not get deprotonated during the transport cycle, but its mutations caused lower uptake rates and enabled the stimulation of transport by Na^+^^18, 20^. These findings suggest that the Na2 site in the APC superfamily represents a remarkably conserved and functionally active cation-binding site. Interestingly, such basic residues are missing from the analogous location in SLC11 transporters, suggesting a distinct proton binding and transport mechanism.

In our current study, we used a distinctive combination of computational and experimental approaches to systematically search for residues that could be involved in functional proton binding and transport in divalent metal ion transporters, as well as to arrive at a plausible mechanism of proton-coupled transport of SLC11 proteins.

## Results

We initially aimed to pinpoint possible proton binding sites where proton binding could have functionally relevant consequences. For this, *in silico* p*K*_*a*_ predictions together with literature data and structural information was used, followed by molecular dynamics (MD) simulations.

### Estimation of side-chain p*K*_*a*_ values

**Figure 1:**
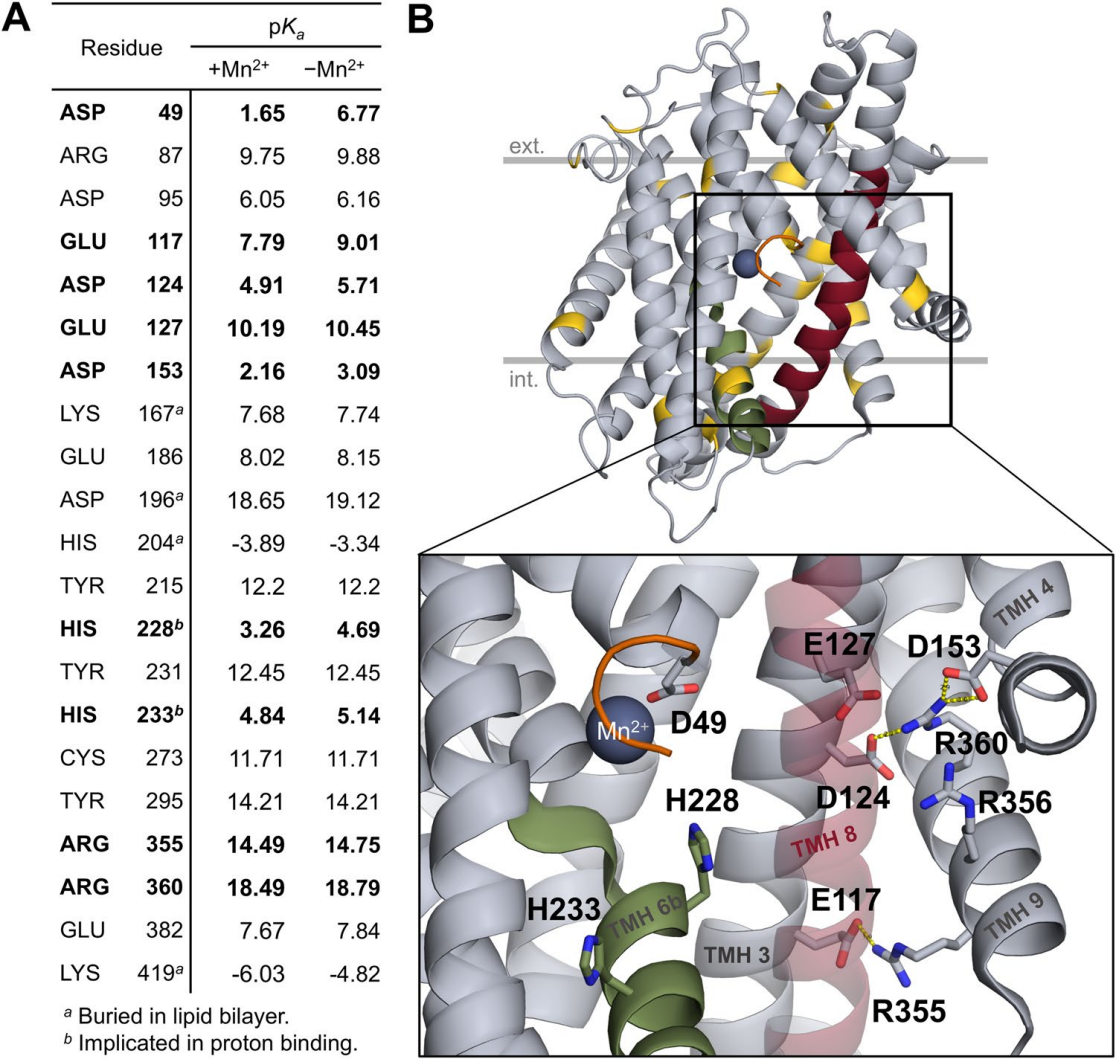
Selected residues based on p*K*_*a*_ analysis and the charged cluster around E127. (**A**) The side-chain p*K*_*a*_ values of titratable residues were estimated *in silico* in ScaDMT, both in the presence and absence of the bound Mn^2+^ ion. Residues having a p*K*_*a*_ shift of more than 1.5 units from their canonical p*K*_*a*_ values in solution are shown. Additionally, H233, mutations of which have been shown to cause pH-independent transport in rat DMT1, is also included for reference. Residues highlighted in bold are shown in the close-up view on the lower half of panel B. (**B**) All residues on panel A are highlighted in gold on the ScaDMT structure. The close-up view of the substrate binding site and a charged residues cluster are shown below. The following regions are color-coded: TMH 1a stub (truncated N-terminus; orange), TMH 6b (green), TMH 8 (red). TMH 8 is shown as a transparent helix in the close-up view.

To assess possible residues that might bind protons during the transport cycle, *in silico* p*K*_*a*_ estimation was performed for a crystallized prokaryotic homologue, ScaDMT^11^. For these calculations, the co-crystallized Mn^2+^ ion was retained, which is the native substrate for ScaDMT. The p*K*_*a*_ of side-chains were estimated in both the presence and the absence of the bound Mn^2+^ ion, in search for side-chain p*K*_*a*_ shifts of 1.5 units or more that would favor protonation (Fig. 1A). Some extreme p*K*_*a*_ values (e.g. D196, H204, K419) for residues that are completely buried in the membrane are likely artefacts arising from the use of the simplified continuum dielectric membrane model and the rigid protein structure. Most of the residues found with high p*K*_*a*_ shifts are on the peripheries of the protein structure, making them less likely to be involved in transport. A marked exception is a series of acidic residues in TMH 3, E127, D124 and E117; forming a cluster of charged residues with R360, R355 and R356 in TMH 9, and D153 in TMH 4 (Fig. 1B), which has been recently suggested to be a possible exit pathway for transported protons^13^. E127 seemed an interesting candidate for a proton carrier residue due to its closeness to the substrate binding site (Fig. 1B) and its relative conservedness in the PFAM Nramp family (60% Glu and 5% Asp in the family sequence alignment). Three further residues were included independently of our p*K*_*a*_ estimations in our subsequent computational studies. D49 is a canonical metal ion binding residue in the SLC11 family^11^, calculated to have a relatively high p*K*_*a*_ value of 6.77 in absence of the Mn^2+^ ion in ScaDMT (Fig. 1A). The conformation of D49 in the ScaDMT X-ray structure can theoretically accept a proton. The conserved histidine residues H228 and H233, shown to cause pH-independent transport in different SLC11 transporters^10, 13^, were also included, despite their low calculated p*K*_*a*_ values of 3.26 and 4.84, respectively (Fig. 1A), which would predict them less likely to directly bind protons at the operational pH of the transporter (5.5–6.5).

### Molecular dynamics simulations of various protonated states

Since binding of the cotransported ion often induces conformational changes in secondary transporters^21–23^, we wanted to know whether proton binding to any of the four selected residues would lead to functionally relevant conformational changes in the protein. To this end, each of the four residues (D49, E127, H228 and H233) was protonated individually *in silico* in ScaDMT and submitted to molecular dynamics (MD) simulations. Conformational changes of the protein were compared to a baseline simulation, which will be referred to as the “canonical system” (i.e. Glu, Asp, Lys, Arg charged; other residues neutral). Five independent simulations of 50 ns each, starting from the same protein conformation were performed for each protonation state. For the MD simulations, due to the absence of either Mn^2+^ and Fe^2+^ parameters in the force-field used, Cd^2+^ was used as a metal ion substrate, which has been shown to bind to the same site as Mn^2+^ and to be transported in ScaDMT^11^.

**Figure 2:**
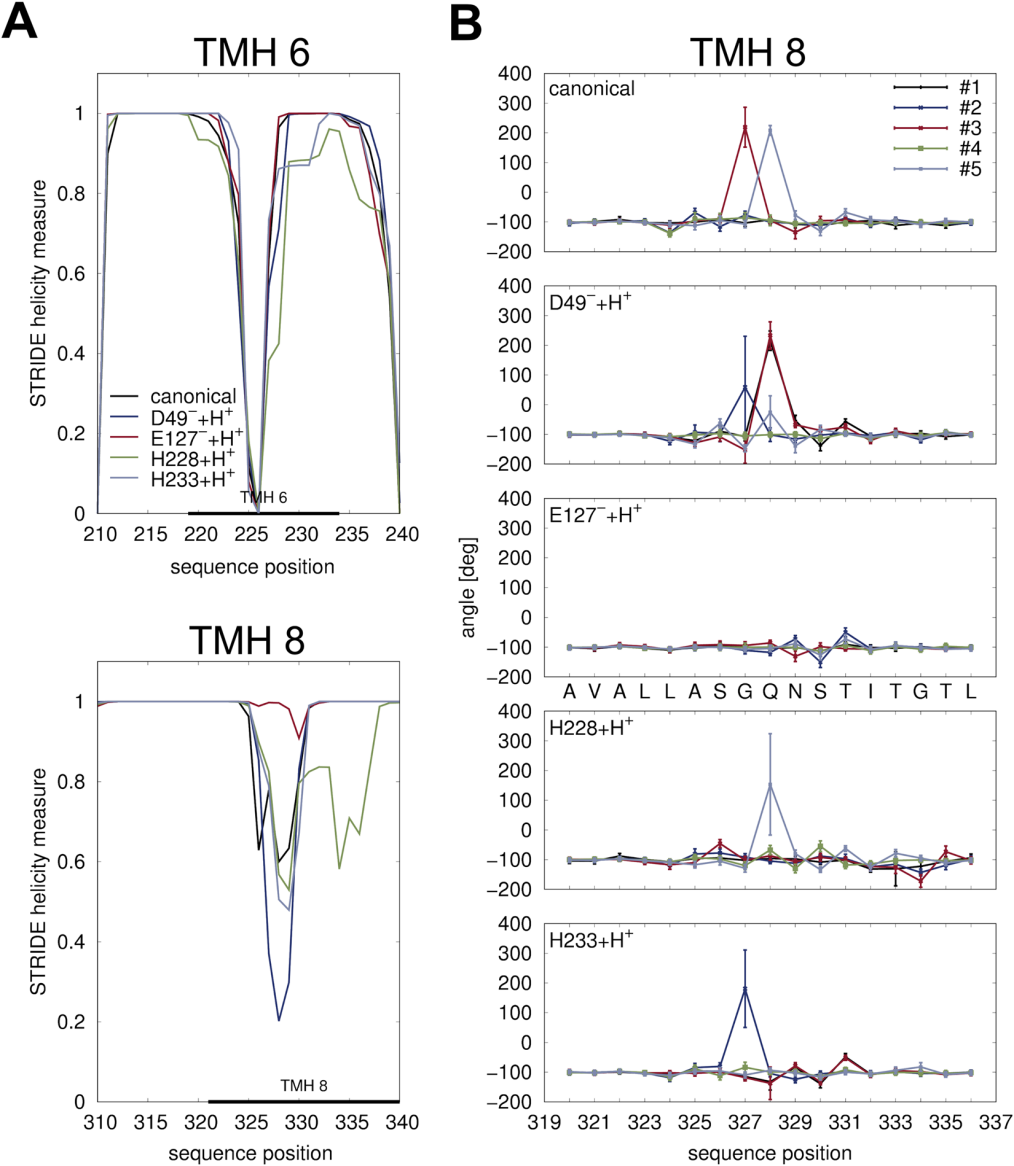
Helicity changes upon protonation in ScaDMT. (**A**) Time average of helicity of each residue as observed in simulations of ScaDMT in various protonation states, quantified using STRIDE^24^. The indicated transmembrane regions were taken from the PDBTM database^62^. Regions with most differences in helicity are shown, which are TMH 6 (top) and TMH 8 (bottom). (**B**) Helicity changes in each MD trajectory were also quantitated by calculating the time average of the sum of dihedral angles *ψ*_*i*_ + *ϕ*_*i*+1_ along the protein chain. For an ideal α-helix, this value is ≈−105°. Large values around 200° correspond to local peptide bond flips.

Quantitating secondary structure content using STRIDE^24^ shows moderate but detectable helicity loss in TMH 6b upon the protonation of H228 and to a lesser extent H233 (Fig. 2A, upper panel). Interestingly, larger helicity changes were observed in region 325–340, which is located in the middle of TMH 8. Helicity is restored upon the protonation of E127, but not any of the three other residues (Fig. 2A, lower panel). A more detailed geometric analysis based on summing two consecutive backbone dihedral angles (*ψ*_*i*_ + *ϕ*_*i*+1_, see Methods for details) shows abrupt changes in backbone geometry near the highly conserved G327 and Q328 residues in the canonical system as well as in several other simulations where either of D49, H228 or H233 is protonated (Fig. 2B). Visual inspection shows that the abrupt backbone angle changes are due to the flipping of the peptide bonds, and the backbone NH groups establishing hydrogen-bonds with the charged E127 side-chain. This effect was not seen in the simulation systems where E127 is protonated.

While residues 325–330 in TMH 8 were modeled as an α-helix in the X-ray structure, they have elevated B-factors compared to the rest of TMH 8, suggesting inherent flexibility. In addition, this region is located near the Na2 site of the Na^+^-coupled leucine transporter LeuT (Supplementary Figure S1A), where cation binding is involved in transport-related conformational changes^17, 25^.

As a next step, we tested whether in addition to E127, other residues would contribute to further stabilizing the structure. To this end, doubly protonated simulation systems were generated, protonating E127 together with each of the D49, H228 and H233 side-chains individually. In the doubly protonated systems, the patterns of secondary structure loss are similar to but less pronounced than those in the singly protonated systems, and the region 325–330 cannot be further stabilized by protonating residues additional to E127 (Supplementary Figure S2).

Interestingly, a glutamic acid residue (E112) also exists in TMH 3 of the leucine transporter LeuT at a location similar to ScaDMT E127, although in a different register on the helix. However, in similar MD simulations of the inward-open LeuT structure (PDB ID: 3TT3), the corresponding region in TMH 8 did not show any instability regardless of the protonation state of E112 (Supplementary Figure S1B), suggesting that the proton-dependent instability of TMH 8 is a phenomenon specific to ScaDMT, and not a general structural feature of the fold family.

### Conformational changes in the intracellular gate region

#### Divalent metal ion binding site

**Figure 3:**
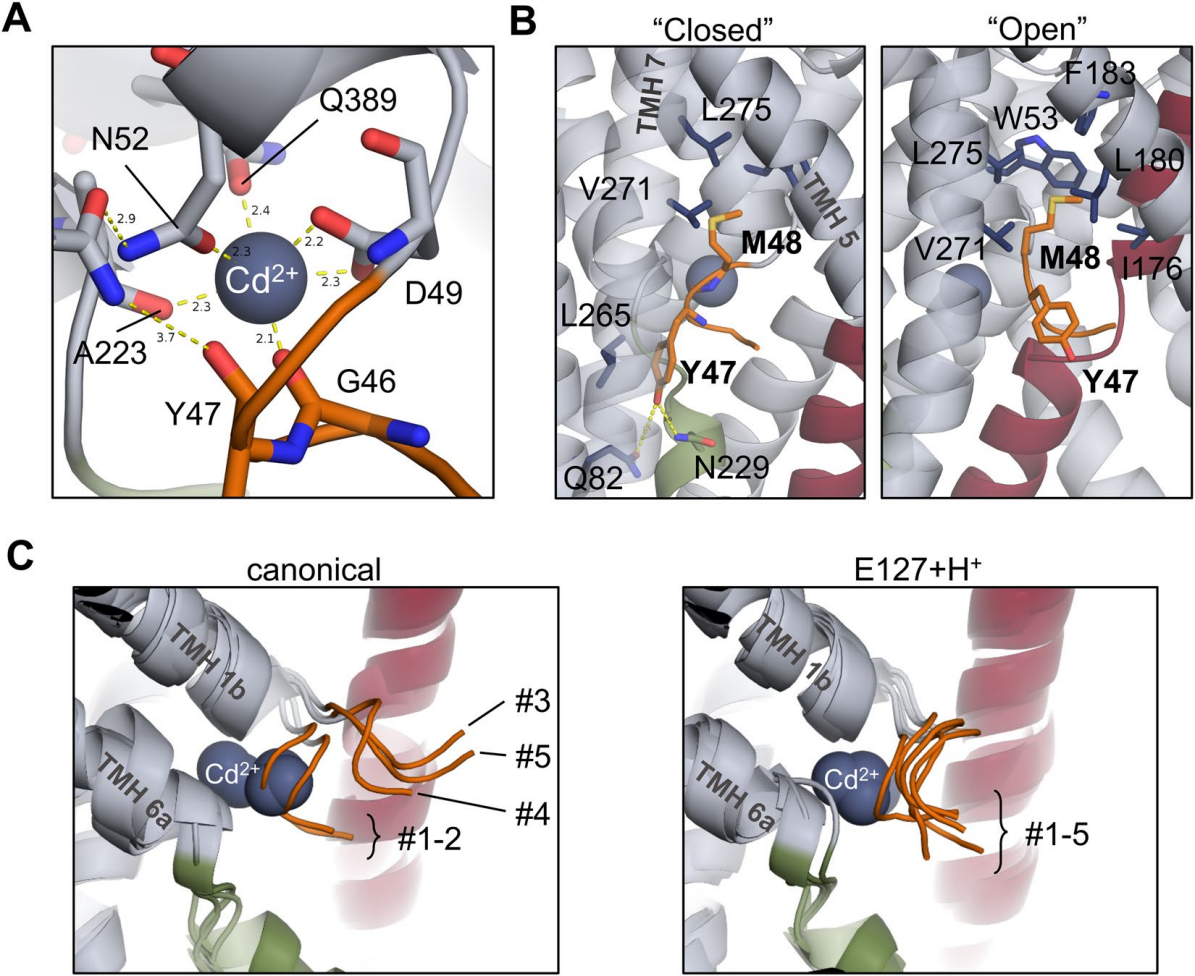
Metal ion coordination and intracellular gate opening in ScaDMT. (**A**) A closed coordination shell of the metal ion substrate evolved in simulations. Compared to the conformation found in the X-ray structure, the backbone carbonyl oxygen of G46 and the side-chain of Q389 fill the remaining two positions in an octahedral coordination shell. (**B**) In the closed conformation (left panel), the side-chain of Y47 fills a cavity between TMHs 6 and 7. When gate opening is observed, M48 enters a hydrophobic pocket formed mostly by TMHs 5 and 7. (**C**) Intracellular gate opening is observed in 3 of 5 “canonical” simulations (left), but not when E127 (right) or any of the residues D49 and H233 is protonated. For clarity, the right panel shows only the results of simulations where E127 was protonated. Gate opening is also observed in 1 of 5 simulations where H228 is protonated (not shown in figure), evolving into a conformation similar to that of canonical #3 and #5.

In some of our simulations, a complete octahedral coordination shell around the bound metal ion substrate emerged, resembling a “closed” state (Fig. 3A). Compared to the ScaDMT X-ray structure, where only 4 of 6 coordination sites are filled, the key rearrangements are the displacement of the Y47 side-chain to fill a previously empty cavity between TMHs 6 and 7, lined by L265, Q82 and N229 (Fig. 3B, left panel), while the backbone carbonyl oxygen atom of G46 comes close to coordinate the bound metal ion. Importantly, on the opposite side of the binding site, Q389, a residue that has not yet been reported to be involved in metal ion binding, approaches to fill the remaining coordination site.

#### Intracellular gate opening

In 3 out of 5 of the canonical simulations (simulations #3, #4 and #5), and in one of the 5 simulations with H228 protonated, we observed that the N-terminal stub of TMH 1a undergoes conformational change reminiscent of gate opening, characterized by the extension of the N-terminal end of the backbone (Fig. 3C, left panel). The Y47 side-chain leaves its pocket between TMHs 6 and 7, and protrudes toward the membrane and the solvent, while the M48 side-chain rotates and extends in the extracellular direction to fill a hydrophobic cavity lined by V271, L275, W53, F183, L180 and I176 (Fig. 3B, right panel). Interestingly, in all other simulations, including all those where E127 was protonated, the intracellular gate remains in the closed conformation throughout the simulations (Fig. 3C, right panel).

### Human DMT1

In addition, we also prepared a homology-based model of human DMT1 (Supplementary Figure S3A, see also Methods). The estimated p*K*_*a*_ values of the analyzed four residues in our model of hDMT1 show comparable values to ScaDMT (Supplementary Figure S3B), including a large p*K*_*a*_ shift for E193, the residue corresponding to E127 in ScaDMT. In analogous MD simulations, the human DMT1 structure also exhibited instability in TMH 8 at the location homologous to that observed in ScaDMT (G412, Q413 in hDMT1), which was dependent on the protonation state of E193 (Supplementary Figure S3C).

In summary, our MD simulations show that the protonation state of E127/E193 correlates with family-specific functionally relevant rearrangements of the protein. Additionally, since we observed that Q389 in ScaDMT becomes a coordinating residue of the metal ion substrate, we reasoned that the homologous residue N472 in hDMT1 might also take part in metal ion binding. To verify the role of these residues in transport function in the human isoform, site-directed *in vitro* mutagenesis was performed and both substrate uptake and electrophysiological properties of the mutant variants were characterized. For the E193 residue, the conservative Asp and the deleterious Ala substitutions were engineered, the E193Q variant was not constructed because the corresponding E112Q mutant in the homologous *E*. *coli* MntH was shown to have greatly reduced transport activity^26^. In addition, the N472A variant of hDMT1 was generated to test the involvement of N472 in substrate binding, while the M294C variant served as a positive control of binding site modification, as M294 is known to take part in metal ion binding^11, 27^.

### Plasma membrane expression and substrate uptake by hDMT1 mutants

**Figure 4:**
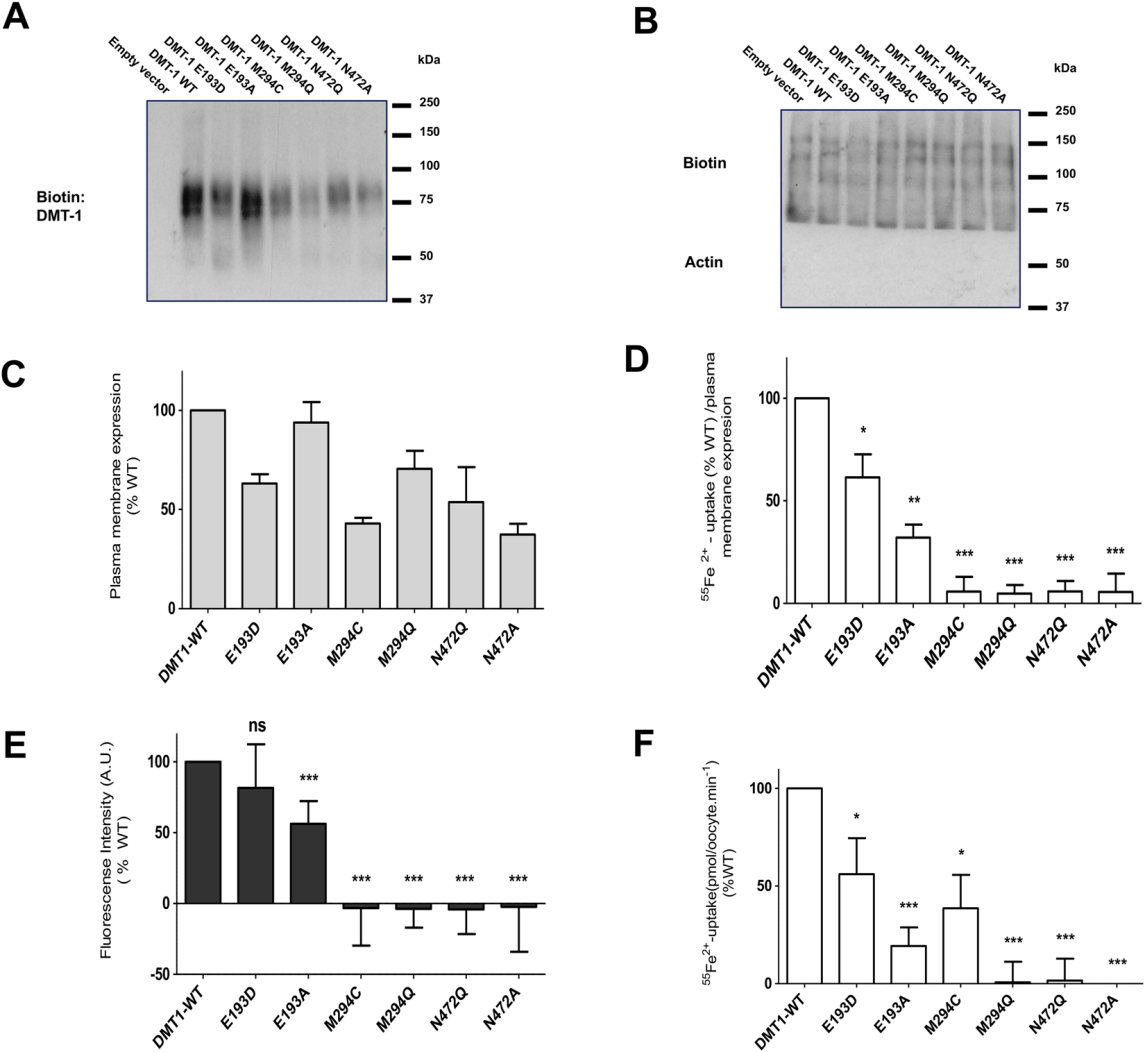
Plasma membrane expression and substrate uptake by hDMT1 and mutants. (**A**) Representative blot of the plasma membrane expression of hDMT1 and the indicated mutants in transiently transfected HEK293 cells. Expression was assessed by immunoblotting of the labeled plasma membrane protein by sulfo-NHS-LC-biotin isolated on streptavidin agarose beds. (**B**) Biotin was used as equal loading control (the absence of actin confirms the purity of the membrane fraction). (**C**) Mean plasma membrane expression of each protein was obtained by averaging the optical density (OD) of the corresponding bands obtained form 3 independent blots. These values were normalized to the OD of its corresponding loading control. (**D**) Radiolabeled iron uptake (^55^Fe^2+^) (1 μM) by HEK293 cells expressing the indicated proteins was measured in uptake buffer at pH 5.5 after 15 min incubation. (**E**) Cd^2+^ (1 μM) transport activity by HEK293 cells expressing the indicated proteins. Changes of fluorescence intensity of Calcium-5-dye were continuously recorded at extracellular pH 6.5 during 10 min incubation with Cd^2+^ and the uptake was determined as the area under the curve. The radioactive iron uptake (**D**) and the fluorescence intensity (E) of each isoform were corrected for the unspecific uptake by empty vector transfected HEK293 cells. Results from 3 independent experiments were normalized to Fe^2+^ (**D**) (N = 41–56) or Cd^2+^ (**E**) (N = 18–24) uptake by hDMT1 WT, corrected according to their plasma membrane expression and represented as the mean ± S.D. (**F**) Uptake of 20 μM ^55^Fe^2+^ (pH 5.5, 10 min) in oocytes expressing hDMT1 and the indicated mutants. Uptake values were corrected for unspecific iron uptake in non-injected oocytes. Data from 6 different batch of oocytes were normalized to the mean iron uptake by hDMT1 WT (0.83 ± 0.3 to 2.79 ± 0.4 pmol∙oocyte^−1^∙min^−1^) and are represented as the mean ± S.D. (25 to 74 oocytes). Statistical differences (**D**–**F**) were assessed using T-test (Fe^2+^-uptake by hDMT1 WT *vs* each mutant); ns p > 0.05; *p < 0.05; **p < 0.01; ***p < 0.001.

Surface biotinylation showed that all the generated mutants were properly expressed in the plasma membrane of transfected HEK293 cells (Fig. 4A and B, expected size 75 kD), although some at lower levels than WT (Fig. 4C). Therefore, in the subsequent experiments using transiently transfected HEK239 cells, all results were normalized to the relative plasma membrane surface expression of each mutant.

In subsequent radiolabeled ^55^Fe^2+^ experiments, no Fe^2+^ uptake was observed for M294 and N472 mutants, while for the E193D and E193A substitutions, uptake was reduced by ≈45 and 80%, respectively, compared to WT (Fig. 4D). Similar results were observed for Cd^2+^ uptake determined by fluorescence measurements (Fig. 4E). Nevertheless, in this case the difference in uptake between WT and E193D was significantly reduced. These experiments were conducted at pH 6.5, due to the loss of sensitivity of the Cd^2+^-sensitive Calcium 5 dye at low pH, which may indicate that E193 mutants have a higher affinity for H^+^ than WT.

Finally, uptake measurements of radiolabeled ^55^Fe^2+^ in *X*. *laevis* oocytes expressing the proteins under study (Fig. 4F) gave similar results as in transfected HEK293 cells, except that the activity of M294C was much higher. However, these experiments were conducted at a higher concentration of non-radioactive Fe^2+^, indicating that this substitution decreases the affinity for Fe^2+^. The lack of functional activity of the N472 mutants points to a key role of this residue in the divalent-metal ion binding of DMT1.

### Fe^2+^ and H^+^ saturation kinetics of E193 and M294 mutants

**Figure 5:**
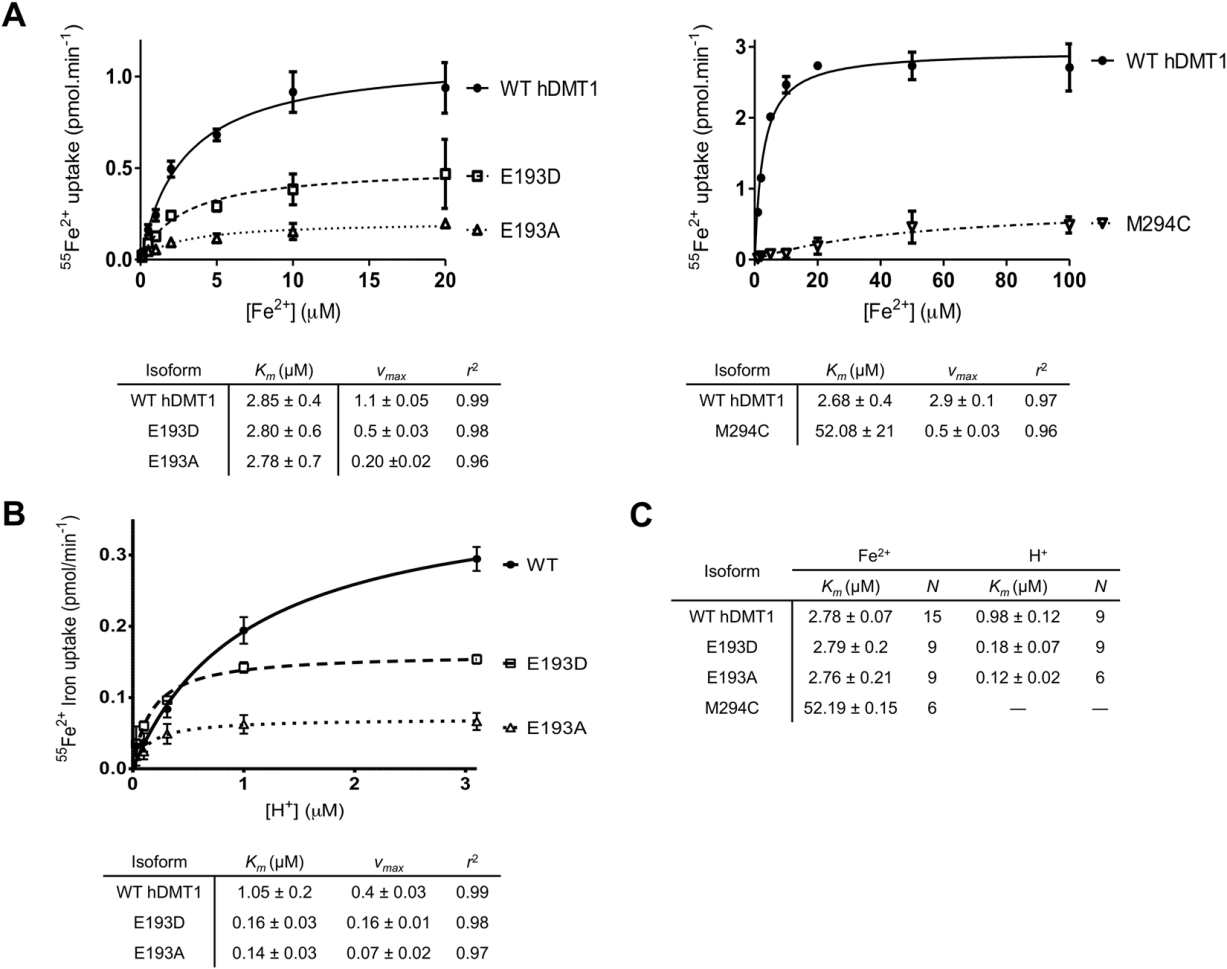
Fe^2+^ and H^+^ saturation kinetics of WT hDMT1 and E193 and M294 mutants in stably transfected HEK293 cells. (**A**) Representative experiments of ^55^Fe^2+^ uptake kinetics by hDMT1 and E193 and M294 mutants as a function of increasing Fe^2+^ concentrations (0.1–20 µM). (**B**) Representative experiment of ^55^Fe^2+^ uptake kinetics by hDMT1 and E193 mutants as a function of increasing H^+^ concentration (pH 5.5–7.5). For each concentration of the substrate, the background uptake measured for the empty vector transfected HEK control cells was subtracted. Uptake values were also corrected according to their plasma membrane expression and represented as the mean ± S.D. A Michaelis-Menten equation (connecting lines) was fit to the data and the obtained kinetic parameters are summarized in a table below each graph. (**C**) Summary table of the apparent affinities for Fe^2+^ and H^+^ of hDMT1 and the indicated mutants. *K*_*m*_ values were calculated from 2–3 independent experiments and are presented as means ± S.D.

To understand the impact of each mutation on the functional properties of hDMT1, the pH-dependence and kinetic properties of Fe^2+^ transport by the mutants showing functional activity were determined. Compared to WT, mutants E193D and E193A exhibited similar affinity for Fe^2+^ (Fig. 5A, left panel). However, the *v*_*max*_ for E193D and E193A were ≈54 and 81% lower than WT, respectively, in line with previous experiments (Fig. 4E,F). In contrast, the substitution M294C showed a drastic decrease in the affinity for Fe^2+^ (*K*_*m*_ = 52.19 µM) (Fig. 5A, right panel; Fig. 5C), confirming the role of this residue in metal ion binding of hDMT1.

As for pH-dependence, the E193D and E193A mutants showed a ≈10-fold increase in the apparent H^+^-affinity compared to WT (*K*_*m*_ = 1.05 µM) (Fig. 5B). For the M294C mutant, Fe^2+^ uptake was only measurable at pH 5.5, and thus, it was not possible to determine the H^+^ saturation kinetics. These findings (summarized in Fig. 5C) are in agreement with the predicted role of residue E193 in the H^+^-hDMT1 interaction.

### Electrophysiological characterization of hDMT1 mutants

**Figure 6:**
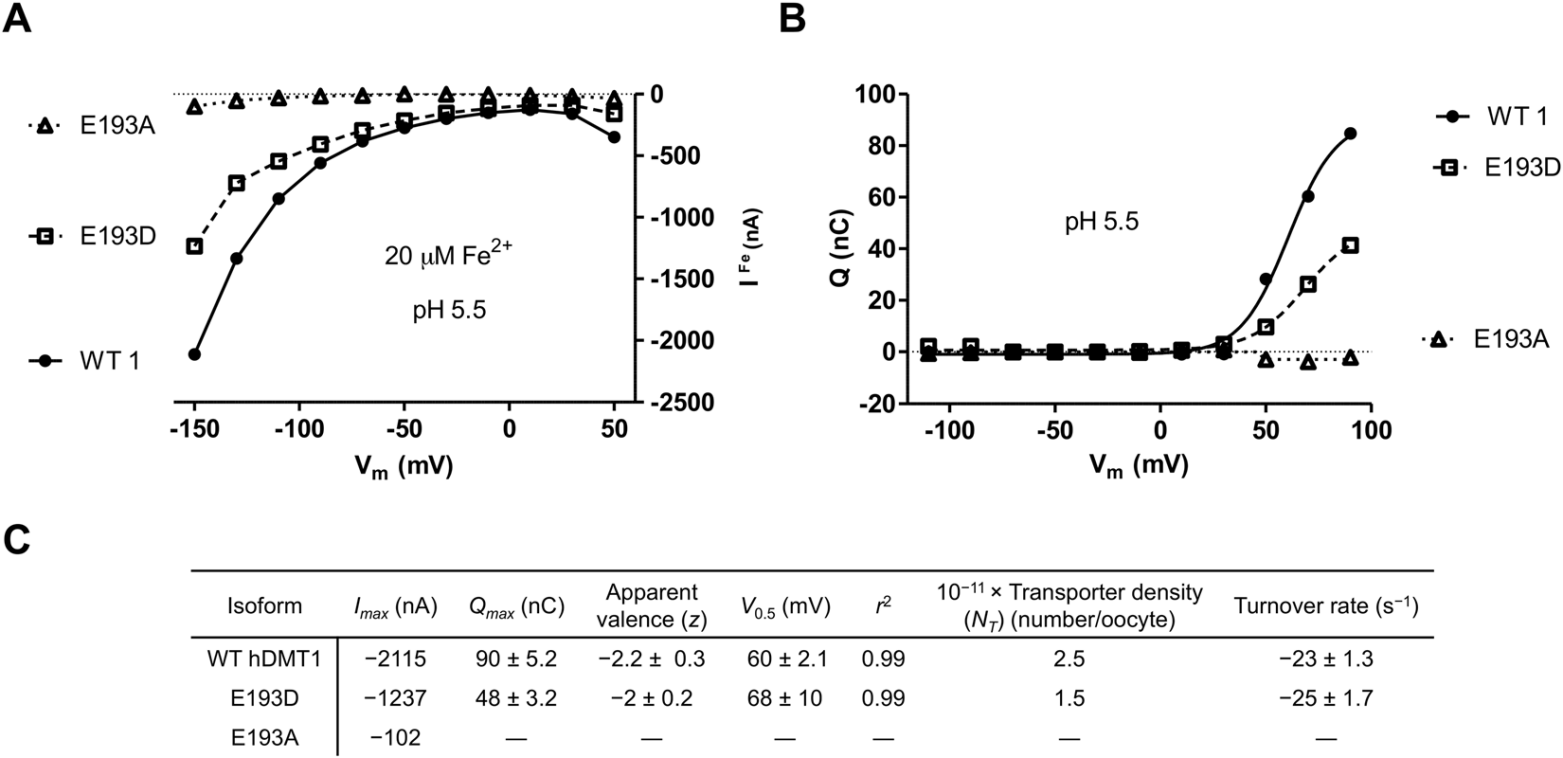
Steady-state and pre-steady state currents for WT hDMT1 and E193 mutants expressed in *X. laevis* oocytes. (**A**) Current-voltage relationship (*V*_*h*_ = −50 mV; pH 5.5; 20 μM Fe^2+^). (**B**) *Q*/*V*_*m*_ relationship (*V*_*h*_ = −50 mV; pH 5.5). Pre-steady state currents were integrated with time to obtain the charge *Q* and depicted at test potentials (*V*_*t*_) ranging from −110 mV to +90 mV. Corresponding curves on panels A and B were recorded in the same oocytes, which were obtained from the same batch. Similar results were observed in 7–10 oocytes from 3 different batches. (**C**) The maximal current evoked by 20 µM Fe^2+^ (Fig. 1A) was taken as *I*_*max*_. Pre-steady state parameters (*Q*_*max*_, *z*, *V*_0.5_ and *r* ^2^) were derived from data in panel B by fitting a Boltzmann equation (connecting lines). The number of functional transporters (*N*_*T*_) expressed in the plasma membrane was determined as *N*_*T*_ = *Q*_*max*_/*z*∙*e* (*e* = 1.6 × 10^−19^ C). Turnover rate was determined as −*I*_*max*_/*Q*_*max*_.

Fe^2+^ evoked inward currents in oocytes expressing WT, E193D and E193A (Fig. 6A) but not M294C, M294Q, N472Q, N472A and in non-injected oocytes (data not shown). Compared to WT, the *I*_*max*_ recorded for E193D and E193A was ≈40 and 95% lower, respectively. Nevertheless, for WT and E193 mutants, Fe^2+^-evoked currents followed a curvilinear relationship with voltage, which increased with the hyperpolarization of the membrane as previously described for hDMT1^28^.

Pre-steady state currents have been previously reported for rat and human DMT1^10, 28^. Compared to WT, the pre-steady state currents for E193D (Fig. 6B) showed ≈45% lower maximal charge transfer (*Q*_*max*_), but similar midpoint (*V*_0.5_ ≈+60 mV), apparent valence (*z*) of the movable charge of ≈−2 and turnover rate of ≈25 s^−1^ (Fig. 6C). The number of functional transporters (*N*_*T*_) was calculated to be ≈40% lower than WT, which also explains the observed ≈45% reduction in *I*_*max*_ and *Q*_*max*_. In contrast, the substitution E193A did not show any pre-steady state currents, which might indicate that the negative charge of the glutamic acid, also present in the E193D mutant, is essential for this property of the transporter. Alternatively, it is possible that our setup was not sensitive enough to detect pre-steady state currents of E193A.

### Electrophysiological properties of the Fe^2+^ and H^+^ saturation kinetics of the E193D mutant

To clarify whether the differences observed between WT and the E193D variant are merely due to reduced expression or mechanistic changes in the transporter itself, saturation kinetics of the electrophysiological properties for both Fe^2+^ and H^+^ were analyzed.

**Figure 7:**
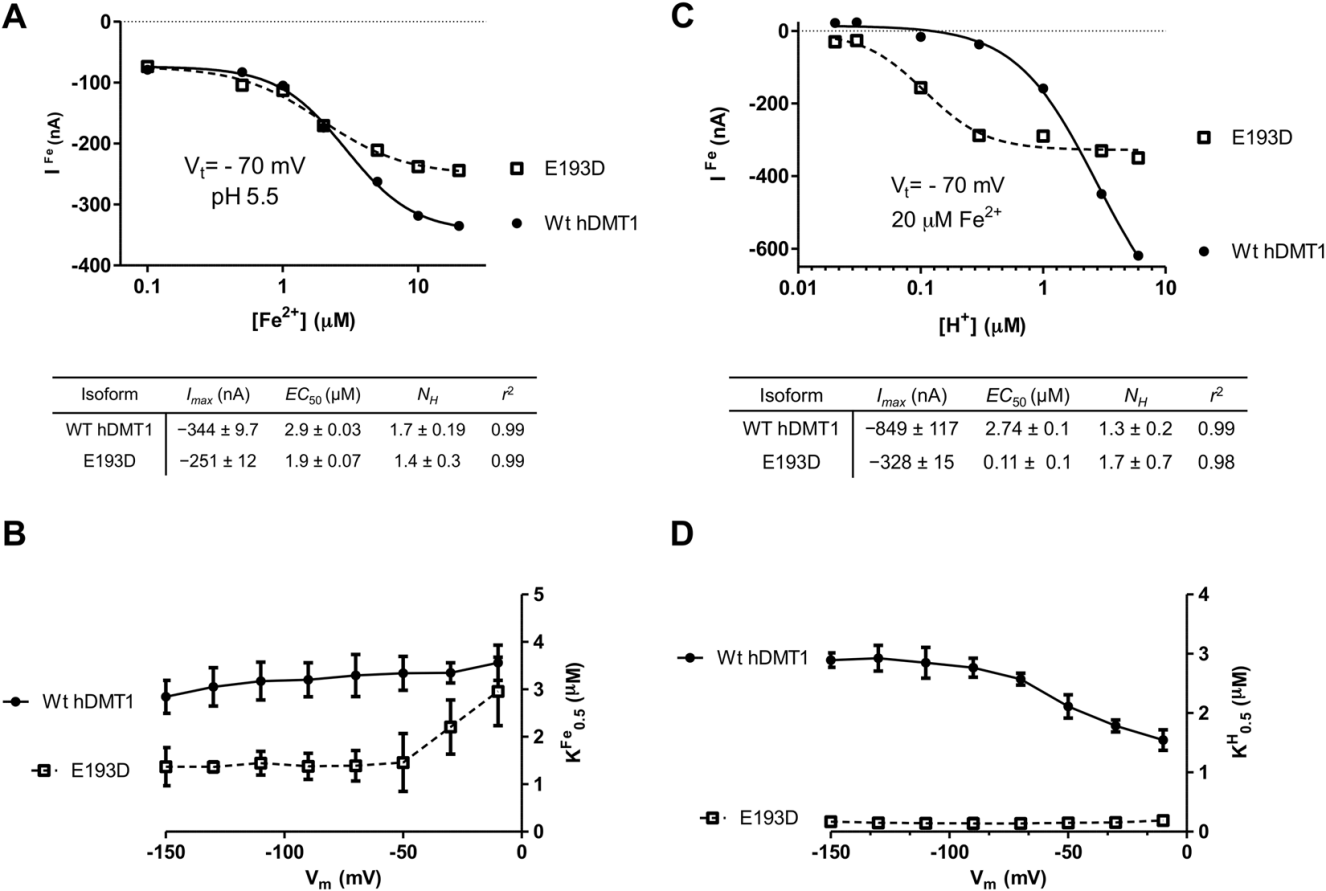
Fe^2+^ and H^+^ saturation kinetics of the steady-state currents of hDMT1 and E193D mutant expressed in *X. laevis* oocytes. (**A**) Representative experiment of Fe^2+^-evoked steady-state currents (*V*_*h*_ = −50 mV; *V*_*t*_ = −70 mV; pH 5.5) as a function of [Fe^2+^]. (**B**) Fe^2+^-transport *K*_0.5_ as a function of membrane potential (*V*_*m*_), each *K*_0.5_ value corresponds to the mean ± S.D. calculated from 4–5 oocytes obtained from 2 different batches. (**C**) Representative experiment of Fe^2+^-evoked steady-state currents (*V*_*h*_ = −50 mV; *V*_*t*_ = −70 mV; 20 µM) as a function of [H^+^]. (**D**) Fe^2+^-transport *K*_0.5_ as a function of membrane potential (*V*_*m*_), each *K*_0.5_ value corresponds to the mean ± S.D. calculated from 4–5 oocytes obtained from 2 different batches. Kinetic parameters of Fe^2+^-transport of the representative panels A and C were calculated by fitting a 4-parameter sigmoidal equation (connecting lines) to the data and are summarized below the corresponding graph.

The Fe^2+^-induced steady-state currents were saturable with Fe^2+^, having a half-maximal (*K*_0.5_) Fe^2+^ concentration for WT and E193D of 2.9 and 1.9 µM, respectively (Fig. 7A). The affinity obtained for WT resembles the one determined in HEK cells (Fig. 5A), while for E193D the *K*_0.5_ for Fe^2+^ is slightly higher. Plotting the *K*_0.5_-*V* relationship (Fig. 7B) showed that WT hDMT1 was modestly voltage-dependent, while E193D showed similar behavior to WT within the range of −150 to −50 mV, and became more voltage-dependent above −50 mV, with *K*_0.5_ values that increase with depolarization, reaching WT values at −10 mV.

Measuring Fe^2+^-evoked currents at different pH values showed that WT was not completely saturable with H^+^, in contrast, the E193D mutant was saturated already at pH 6.5, as also reflected by the highly significant decrease in the apparent *K*_0.5_ for H^+^ (≈0.11 µM) (Fig. 7C), being in agreement with our previous results (Fig. 5B and C). Regarding the *K*_0.5_-*V* relationship (Fig. 7D), WT hDMT1 showed a moderate increase in the apparent *K*_0.5_ for H^+^ with hyperpolarization. Strikingly, in E193D it was completely voltage-independent, indicating that the ion well effect^10^ is lost, likely because the ion binding site is more exposed. Altogether these results reflect a change in the transport mechanism of the E193D mutant, probably due to the mutagenesis-induced conformational changes of the protein, rather than protein expression.

Regarding the Hill coefficient (*N*_*H*_) for H^+^ and Fe^2+^ (Fig. 7A and C), which reflects the cooperativity among multiple ligand-binding sites, it was in both cases ≈1.5 and didn’t show voltage-dependence (data not shown), which suggests that there is no change in the stoichiometry of the transport process due to the mutation, or at least, in the number of allosteric binding sites. In line with this observation, the H^+^-driven Fe^2+^-induced currents and the Fe^2+^-transport by the E193D mutant (Figs 4F and 6A, respectively) were reduced to the same extent, which further supports the idea of an unaltered stoichiometry due to the mutation.

### Electrophysiological characterization of the H^+^-leak mediated by E193 mutants

Since the E193 mutants significantly modified the interaction of DMT1 with H^+^, the impact of this mutation on the DMT1-mediated H^+^-leak^10, 28^ in the absence of metal ion was studied. For both WT and E193 mutants, H^+^-leak currents followed a curvilinear relationship with voltage, and increased with the hyperpolarization of the membrane (Supplementary Figure S4A). *I*-*V* relationships for WT and E193D were almost identical, while currents in the E193A mutant were similar but much lower. The H^+^-leak currents for both WT and E193D mutant were saturable, with similar kinetic parameters and voltage-dependence (Supplementary Figures S4B and C), but for E193A, they were only measurable at the lowest pH, so the kinetic parameters could not be determined. These results indicate that the position E193 does not seem to play role in the H^+^-leak through hDMT1. Nevertheless, since the H^+^-leak currents were significantly reduced with the substitution E193A, this could reflect the overall loss of activity of this variant or conversely the relevance of the negative charge, present in both WT and E193D mutant, to this particular feature of the transporter.

### Dependence of pre-steady state currents on [H^+^]

**Figure 8:**
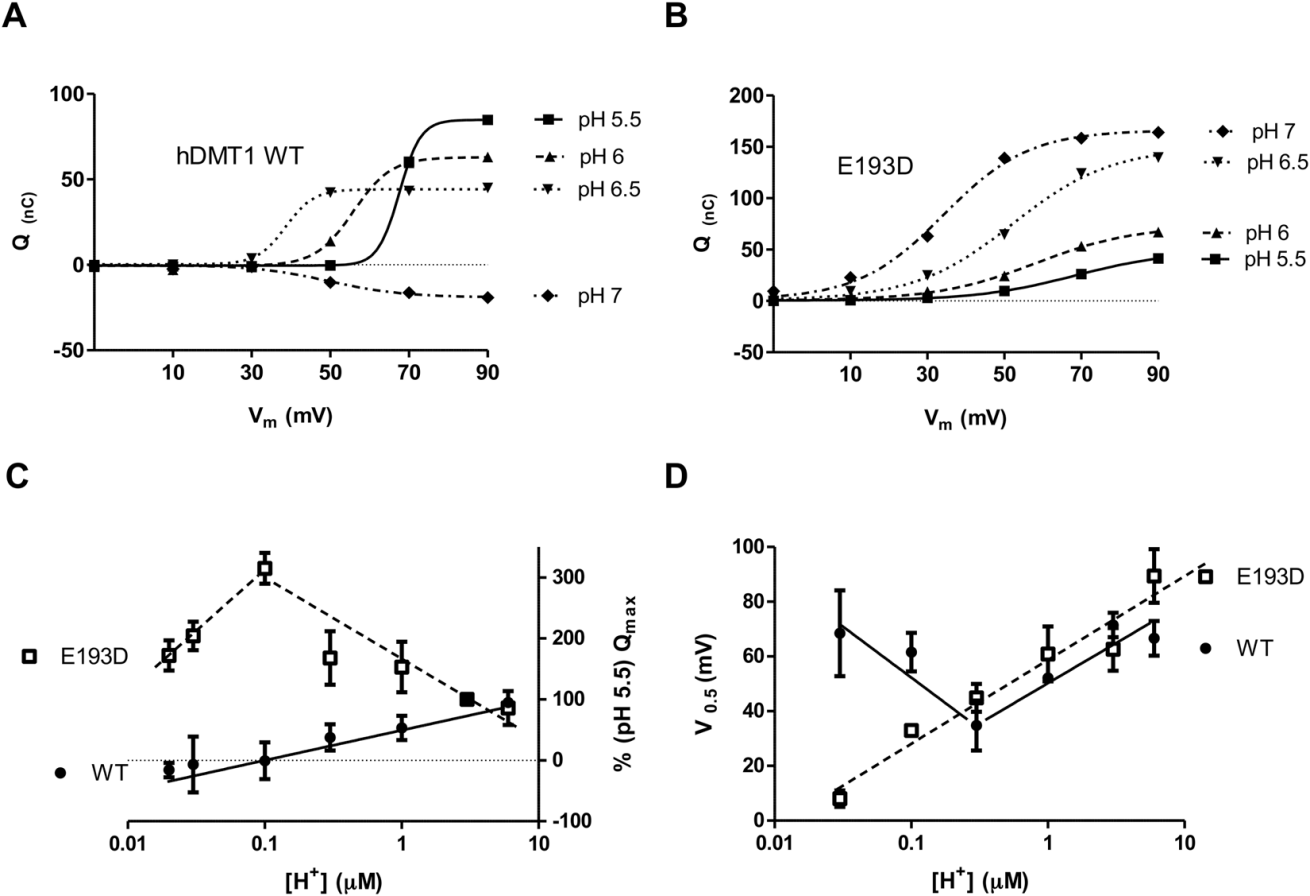
pH-dependence of the pre-steady state currents of hDMT1 and E193D mutant expressed in *X. laevis* oocytes. Dependence of the *Q*/*V*_*m*_ relationship of hDMT1 WT (**A**) and E193D (**B**) mutant on [H^+^]. Pre-steady state currents were integrated with time to obtain the charge *Q*, and depicted at test potentials *V*_*t*_ (−10 mV to +90 mV) from the holding potential *V*_*h*_ = −50 mV at the indicated pH values. A Boltzmann equation was fit (connecting lines) to data in panels A and B to obtain the kinetic parameters (*Q*_*max*_ and *V*_0.5_). (**C**) Dependence of *Q*_*max*_ on [H^+^]. For each individual oocyte, *Q*_*max*_ values were normalized to the *Q*_*max*_ determined at pH 5.5 (WT *Q*_*max*_ 81.6 to 97.7 nC; E193D *Q*_*max*_ 41.55 to 81.6 nC). Represented *Q*_*max*_ values correspond to the mean ± S.D. calculated from 5–13 oocytes obtained from 4 different batches. (**D**) *V*_0.5_ as a function of [H^+^], each *V*_0.5_ value corresponds to the mean ± S.D. calculated from 3–10 oocytes obtained from 4 different batches. ﻿Connecting lines in panels C and D were drawn only for illustration purposes.

To better understand the role of the position E193 in the H^+^-DMT1 interaction, the dependence of the pre-steady state currents on external H^+^ concentration was studied. For the WT, the maximal charge transfer (*Q*_*max*_) increased linearly with increasing external concentration of H^+^ (Fig. 8A and C), and the voltage for maximal charge midpoint (*V*_0.5_) decreased with decreasing [H^+^] until the *Q*_*max*_ switched to negative values at pH 6.5 (Fig. 8A and D). Interestingly, contrary to WT, for the E193D mutant the *Q*_*max*_ increased linearly with the reduction of the external concentration of H^+^ until pH 6 (Fig. 8B and C) and showed positive values for the complete pH range tested (Fig. 8C). Conversely, similar to WT, the voltage for maximal charge midpoint (*V*_0.5_) decreased with decreasing external concentration of H^+^ (Fig. 8D). Nevertheless, in contrast to WT, the *V*_0.5_ did not start to increase again at pH 7, indicating a different voltage-dependence. Pre-steady state currents of DMT1 are consequence of the dissociation of the H^+^ from the transporter and reorientation of the empty charged protein within the plasma membrane. Therefore, the difference observed between WT and E193D might reflect a different arrangement of the H^+^ binding site, additionally, the decrease of the *Q*_*max*_ values with the increase of [H^+^] observed for the E193D mutant could be consequence of a slow dissociation of the H^+^-DMT1 complex due to the high affinity of the mutant for H^+^ (*K*_0.5_ ≈ 0.1 µM).

### Accessible rotameric states

Overall, the altered H^+^-affinity, voltage-dependence and the drastic change in the pre-steady state currents of E193 mutants prompted us to assess protonation-dependent changes in the microscopic conformation of the E127 side-chain in our ScaDMT simulations in order to reveal possible proton-transport mechanisms.

**Figure 9:**
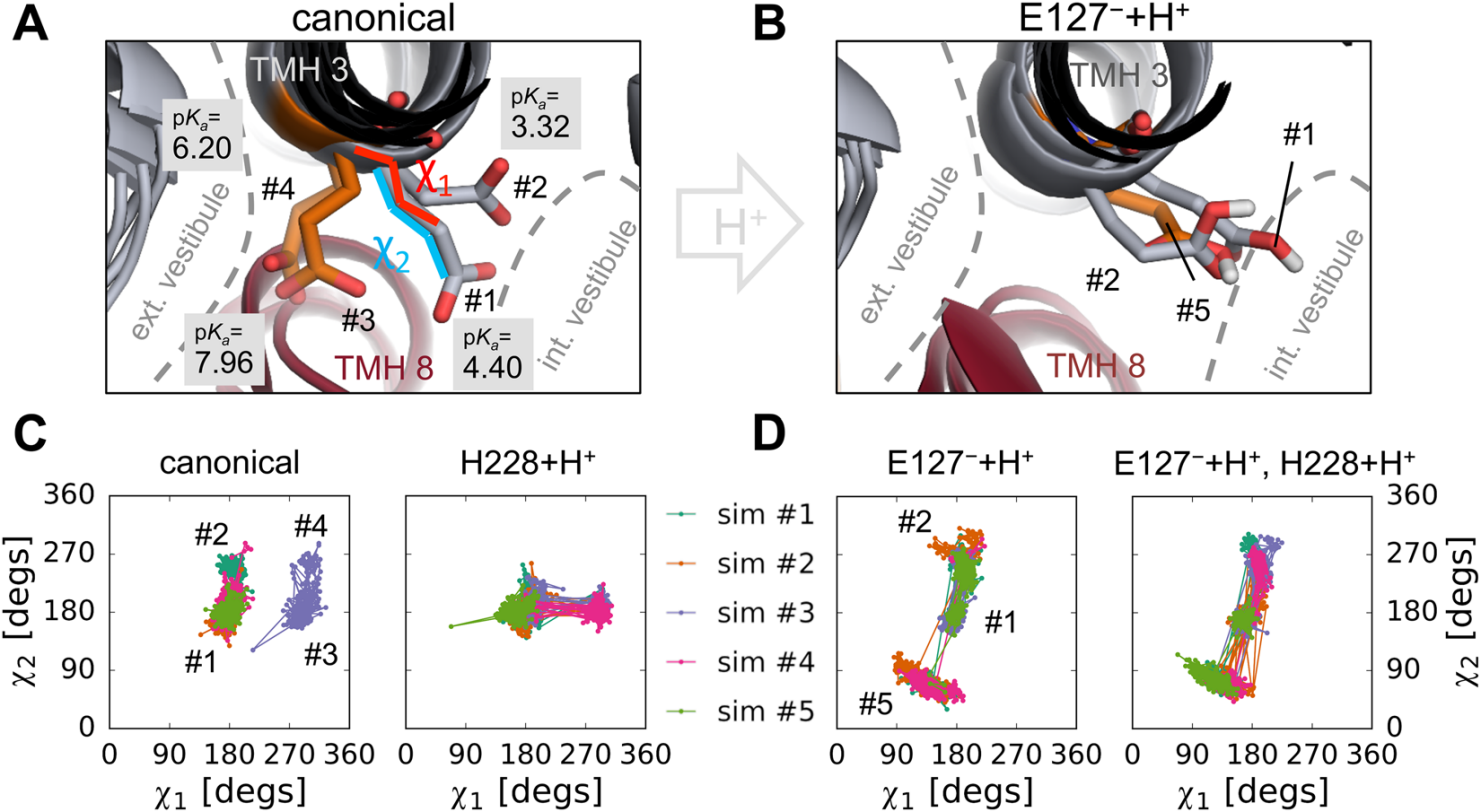
Accessible rotameric states of E127 in ScaDMT. Cluster centroid conformations of the E127 side-chain observed in the “canonical” simulations (**A**) and when E127 is protonated (**B**). Accessibility of the extracellular and intracellular vestibule, cluster numbers, the *χ*_1_ and *χ*_2_ angles of the side-chain, and side-chain p*K*_*a*_ values in each conformation are indicated. The five simulation trajectories are shown color-coded for each protonation state (**C**,** D**), mapped into the space of the *χ*_1_-*χ*_2_ dihedral angles.

Analysis of the accessible rotameric states of the E127 side-chain, as described by the *χ*_1_ and *χ*_2_ dihedral angles, during the MD simulation trajectories shows four major conformational clusters in the canonical system (marked #1-#4, Fig. 9C). Clusters #1 and #2 correspond to a conformation that is oriented toward a solvated vestibule accessible from the intracellular side. In contrast, conformations in clusters #3 and #4 are expected to be in contact with the extracellular medium through the substrate binding site (Fig. 9A). Protonating D49 or H233 does not significantly change the pattern of accessible states (data not shown). However, protonating H228 enhances the transitions between clusters #1 and #3, possibly indicating a lowered free energy barrier between these two states (Fig. 9C, right panel). When E127 is protonated, clusters #3 and #4 become completely inaccessible, indicating that the preferred orientation of the protonated E127 side-chain is toward the intracellular medium (Fig. 9D, left panel). In addition, a new distinct cluster, marked as cluster #5, appears, corresponding to a conformation where the added proton is pointing “upwards” toward the extracellular side (Fig. 9B). Transitions between clusters #1, #2 and #5 are also enhanced by the additional protonation of H228 (Fig. 9D, right panel).

Additionally, p*K*_*a*_ estimation of the E127 side-chain in the centroid conformations of the four clusters in the canonical simulations reveals a higher p*K*_*a*_ value in clusters #3 and #4 (7.96 and 6.20, respectively) compared to clusters #1 and #2 (4.40 and 3.32, respectively), indicating that proton binding is more likely in the extracellularly accessible conformational clusters (Fig. 9A). Taken together, these results hint at a possible inward-rectified mechanism of proton transport by the E127 side-chain.

### Propagation of signal for gate opening

Since no systematic changes in the hydrogen bonding pattern could be observed between closed and open states of the intracellular gate in our MD simulations, the signal for gate opening upon proton loss is likely not mediated by hydrogen bonds. Alternatively, to test whether the signal is the loss of the electrostatic charge of the proton itself, we prepared an unphysical simulation system of ScaDMT carrying an E127 side-chain that is deprotonated but uncharged. In the resulting five MD simulations of this system, no loss of helicity throughout TMH 8 (Supplementary Figure S5A) and a closed intracellular gate were observed, similarly to the protonated E127 system. However, the neutral E127 side-chain displayed marked flexibility and could access all of the states of either the deprotonated or the protonated E127 side-chain (Supplementary Figure S5B). Thus, proton release likely triggers gate opening directly through Coulombic interactions between the E127 side-chain and the gate, while the presence of the H^+^ ion, and not only its charge is required to stabilize the E127 side-chain in the low-p*K*_*a*_ orientation.

### E193D and E193A variants of hDMT1

To establish a link between our experimental results of the hDMT1 E193 mutants and our computer simulations, five simulations for each of the protonated and deprotonated E127D (ScaDMT) and the E127A variant were set up, which all exhibited a highly stable TMH 8 (Supplementary Figure S6A, left panel) and no observable intracellular gate opening. The results were reproduced in simulations of the human DMT1 model carrying the corresponding E193D and E193A mutations (Supplementary Figure S6A, right panel). Conformational analysis showed that only a single conformational cluster is present for the D127/D193 side-chain in E127D/E193D mutants, which is facing the intracellular vestibule (Supplementary Figure S6B). The calculated p*K*_*a*_ values of the Asp side-chains in these conformations were between those calculated for the extra- and intracellularly facing conformations of E127 in ScaDMT, being 5.33 and 4.60 pH units for D127 in ScaDMT and D193 in hDMT1, respectively (Supplementary Figure S6B). We propose that these changes in conformational stability and proton affinity are linked to the observed changes in pre-steady state charge movements and experimental proton affinity between the wild-type and the E193D variant.

## Discussion

Applying a combination of computational and experimental methods, our efforts have highlighted two novel residues as important determinants of function.

Based on our MD simulations, Q389 in ScaDMT (N472 in hDMT1) is a novel substrate-binding residue in divalent metal ion transporters. Mutagenesis experiments confirmed that altering the length of N472 in hDMT1 (N472Q) and removing the side-chain altogether (N472A) has detrimental effects on iron uptake, similarly to M294Q. In contrast, the M294C variant retains metal ion binding, although with significantly decreased affinity, reinforcing its role in substrate binding^11, 27^, while not affecting transition metal selectivity, as our radioactive ^55^Fe^2+^ and Cd^2+^-sensitive fluorescence uptake experiments suggest. A comparison of inward- and outward-open states of the leucine transporter LeuT (PDB IDs: 3TT3, 3TT1) suggests that coordination of the substrate by Q389/N472 might be the step required for closure of the substrate binding site on the extracellular side, drawing TMH 10 and TMH 1b close to each other.

While the unusually high p*K*_*a*_ value of the E127/E193 side-chain and the significant stabilizing effect of its protonation alone might argue in favor of E127/E193 being constantly protonated, mutant variants at position 193 in hDMT1 show altered proton binding as well as transport properties, indicating that proton binding is dynamic at this position and is also affecting transport. In addition, we calculate a significantly lower p*K*_*a*_ value for E127/E193 for conformations in our MD simulations (Fig. 9A) compared to the crystallized conformation. Due to the presence of the lipid bilayer and room temperature conditions, the MD simulations might reflect the physiological state of the protein better than the crystallized conformation, and thus the decreased p*K*_*a*_ values would support the presence of conformations where proton dissociation from E127/E193 can happen. Since protons are fixed to the protein in MD simulations, we cannot observe proton dissociation during our simulations. However, this does not rule out the possibility that proton hopping from E127/E193 to a nearby side-chain or water molecule would be energetically favorable.

There are several pieces of evidence suggesting that the E193D variant has increased binding affinity for protons. Apart from higher apparent affinity in measurements of uptake transport kinetics (Fig. 5B and C), the E193D variant showed saturation of current already at pH 6.5 (Fig. 7C), likely because the protein cannot mediate higher transport-dependent uncoupled currents. In line with these findings, we found a highly significant increase of *Q*_*max*_ compared to WT at the lowest H^+^ concentration tested (pH 7.7), indicating that negatively charged residues responsible to bind H^+^ are mostly outward-facing in E193D, since the reorientation of the empty transporter is expected to be the main cause of the pre-steady state currents under high pH conditions. Interestingly, at high pH (7.7–7.0) the *Q*_*max*_ values increased with [H^+^] (Fig. 8C), which is the expected behavior for Na^+^-coupled symporters such as SGLT1^29^, corresponding to the ability of the transporter being able to bind and unbind more ions as they become available. However, at lower pH (pH 6.5–5.2), *Q*_*max*_ gradually decreased with the increase of [H^+^], reminiscent of inhibitor phlorizin binding to SGLT1, which has been shown to lock the transporter in an electroneutral state^29, 30^. Similarly, we speculate that increasing proton concentrations could lock the E193D variant in an occluded, uncharged conformation. Hindered loss of proton due to higher affinity in turn would affect intracellular gate opening, as suggested by our molecular dynamics simulations. This idea is supported by our p*K*_*a*_ calculations (Supplementary Figure S6B) showing a lower p*K*_*a*_ for D193 than E193 in an inward-facing orientation, and the fact that the E193D mutant shows a 10-fold increase in *K*_0.5_ for H^+^, but diminished *v*_*max*_ and *I*_*max*_ parameters (Figs 5C and 6C). Additionally, the reduced flexibility of the D193 side-chain as seen in our rotamer analysis could explain the voltage independence of the apparent H^+^ binding affinity (*K*_0.5_) and the altered pre-steady state current pattern by removing the rate-limiting step of side-chain reorientation.

Strikingly, the E193A variant under our hands is able to mediate iron uptake that is about 30% of wild-type with a significantly reduced current in our electrophysiology setup. Since most of the transport-related current in wild-type hDMT1 is expected to be transport-dependent uncoupled proton currents^9, 10^, it is likely that E193A is unable to host such uncoupled currents. The fact that we still see iron transport is nevertheless compatible with previous results showing that iron uptake is possible without proton transport^10^. Interestingly, proton concentration can still stimulate iron transport of the E193A mutant to some extent between pH 6–7.5 (Fig. 5B). We believe this can be attributed to other protonatable residues in hDMT1 that could contribute to its transport properties, for example, by stabilizing the protein structure, even though these bound protons might not themselves be transported. Residues in Fig. 1A that have a high p*K*_*a*_ shift and lie outside of the transmembrane region could be potential examples, as many of them are also conserved in human DMT1.

**Figure 10:**
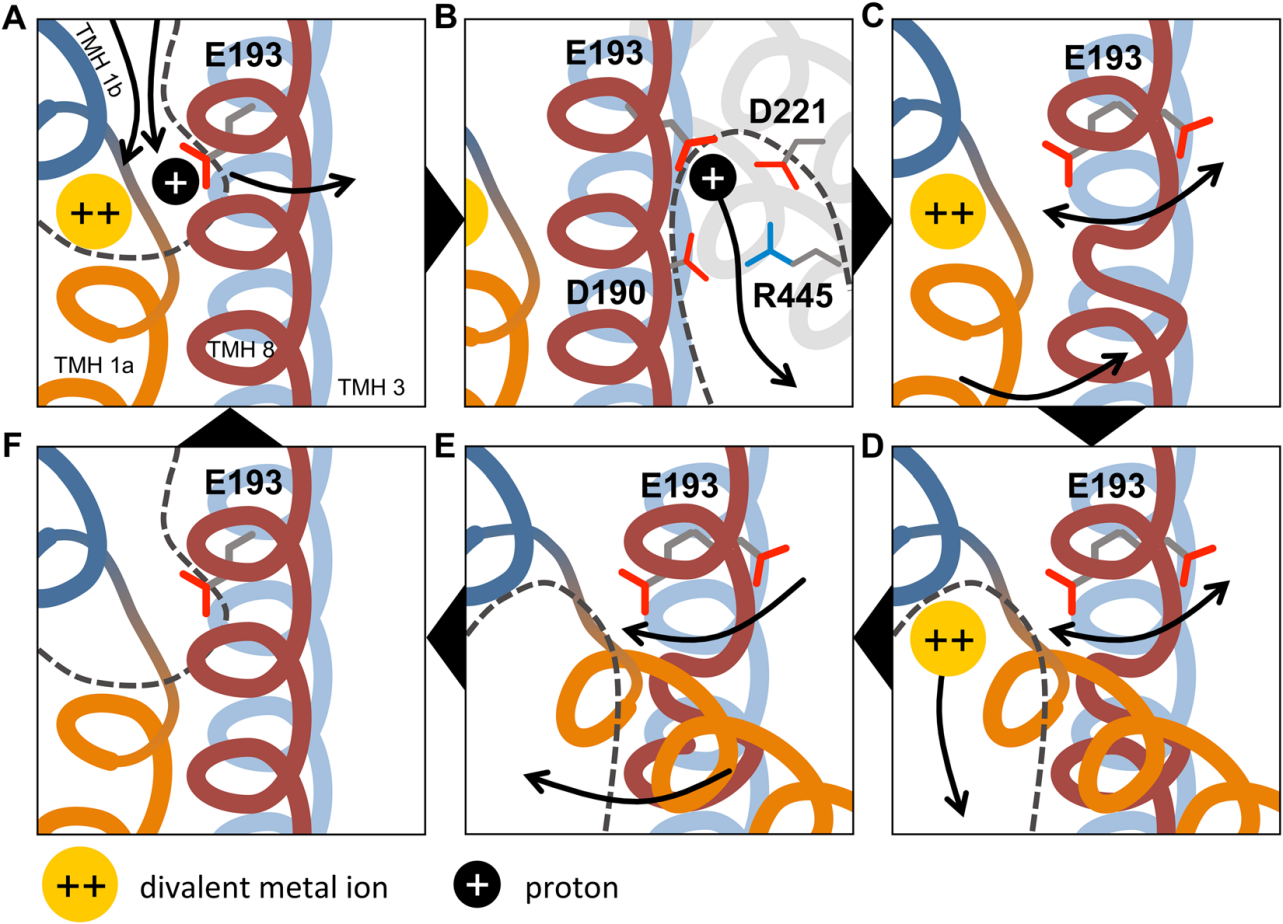
Proposed mechanistic model of the transport cycle of hDMT1. Transmembrane helices are shown color-coded: TMH 1a (orange), TMH 1b (dark blue), TMH 3 (light blue), TMH 8 (red), with TMH 3 located behind TMH 8. Divalent metal ion substrate is shown as yellow disc; proton as black disc. Water-accessible vestibules are indicated with dashed lines. Arrows indicate movements of components. (**A**) Substrate binding happens in the high-p*K*_*a*_ state of E193, likely in an ordered manner. After substrates bind, a conformational switch is proposed to occur, bringing the protein into an inward-open-like occluded state, where E193 flips over to a low-p*K*_*a*_ conformation. (**B**) In the low-p*K*_*a*_ conformation, proton dissociation occurs possibly along a pathway flanked by D221, D190 and R445 (D153, D124 and R360 in ScaDMT, respectively). (**C**) The loss of the proton induces three key conformational changes: the partial unwinding of TMH 8 near residues 410–417 (corresponding to residues 325–330 in ScaDMT), increased flexibility of the E193 side-chain and the opening of the intracellular gate. At this point, substrate-dependent uncoupled proton leak could happen at high proton concentrations by jumping back to state A, if transition to state D is slow enough. (**D**) After gate opening, the metal ion substrate is solvated and leaves the binding site. (**E**) Local helix unwinding in TMH 8 facilitates the recovery of E193 into the high-p*K*_*a*_ state, and closure of the intracellular gate happens. (**F**) The high-affinity outward-open state is recovered. Additionally, this mechanism also allows for the uniport of metal ions in the absence of protons through cycle A-D-E-F-A.

A possible mechanism of how proton binding to E193 could affect substrate transport in hDMT1 is suggested by our molecular dynamics simulations. Our p*K*_*a*_ estimations indicate that compared to the other studied residues (D49, H228 and H233), the E127 residue is the most likely proton acceptor near the substrate binding site. The presence of the proton helps lock the E127 side-chain in a low-p*K*_*a*_ conformation to facilitate the dissociation of the proton. We suggest that allosteric coupling between the E127/E193 side-chain and the intracellular gate is established through Coulombic interactions, and the trigger signal for gate opening is the loss of the charge from the E127 side-chain. The cluster of charged residues (D153, R360, D124 in ScaDMT; D221, R445, D190 in hDMT1) constitutes a possible exit pathway of the proton into the water-solvated intracellular vestibule. After proton dissociation, the local unwinding of TMH 8 might be necessary to sterically aid the reorientation of the E127/E193 side-chain into a high-p*K*_*a*_ proton-accepting state. The overall suggested mechanism is summarized in Fig. 10. It should be noted that there are still several details of the proposed mechanism that need to be investigated, including how proton dissociation from the E127/E193 side-chain happens under transporting conditions, and which molecular events trigger this step. Therefore, further experiments will be conducted in the future to clarify these open questions.

While we provide a plausible mechanism of proton-coupled transport, we cannot rule out the possibility that other residues are also involved in proton coupling. In particular, protonation of H228 in ScaDMT simulations is able to trigger intracellular gate opening, however, only when E127 is deprotonated, which is unlikely, due to the consistently high side-chain p*K*_*a*_ of E127 and the stabilizing effect of its protonation on TMH 8. Intolerance of the structure to a protonated H228 side-chain might be due to the low proton affinity of the protein conformation captured in the crystal. However, protonation of H228 in MD simulations with ScaDMT affects the conformation of E127, indicating intimate cross-talk between the two residues (Fig. 9C and D). Finally, it should be taken into account that the R356 residue in ScaDMT, proposed to be part of the proton exit pathway (Fig. 1B), is not conserved in human DMT1. Therefore, we expect species-specific differences between the role and extent of E127/E193-mediated proton transport and also the transport mechanisms themselves.

In summary, our current work provides the first mechanistic insight into the proton coupling and transport mechanism of divalent metal ion transporters. A previously uncharacterized residue, E193 in hDMT1, is proposed to translocate protons in an inward-rectified manner by alternating contact with the solvent on each side of the membrane bilayer. This mechanism is unique and not akin to currently known proton cotransport mechanisms in similar APC-fold transporters. Our study also complements the X-ray structure by identifying N472, which is predicted to be a novel metal ion substrate binding residue in SLC11 divalent metal ion transporters.

## Methods

### p*K*_*a*_ calculations

The side-chain p*K*_*a*_ values were calculated in two steps based on similar work on the leucine transporter LeuT^31^. First, PROPKA 3.1^32, 33^ was used to estimate side-chain p*K*_*a*_ as if the protein was solvated in water, second, APBSmem^34^ was used to calculate the p*K*_*a*_ shift due to the presence of the membrane bilayer. The calculation was performed both in the presence and the absence of the bound Mn^2+^ ion in ScaDMT. For PROPKA 3.1, default options were used. For APBSmem, the PARSE force-field was used, 0.1 M monovalent counterions with 2.0 Å radius, 298.15 K temperature, and the dielectric constants of protein, solvent, membrane and headgroup were set to 2.0, 80.0, 2.0 and 80.0, respectively. Membrane and headgroup thickness of 42.0 Å and 9.0 Å were used, respectively, with the membrane bottom at −21.0 Å. Calculations were performed with the Npbe method, Spl2 charge model and three-step grid refinement as described previously for LeuT^31^.

### MD simulations and trajectory analysis

Coordinates of the inward-open ScaDMT were taken from the Orientations of Proteins in Membranes database^35^ (PDB ID: 4WGW). The co-crystallized manganese﻿ ion (Mn^2+^) was changed to cadmium (Cd^2+^) due to the lack of parameters for Mn^2+^ in the CHARMM36 force-field. The choice of Cd^2+^ is supported by the fact that it is a substrate for both ScaDMT and hDMT1^8, 11, 36^. The protein was embedded into a POPE (palmitoyl-oleoyl-phosphatidyl-ethanolamine) bilayer for ScaDMT, or POPC (palmitoyl-oleoyl-phosphatidyl-choline) bilayer for hDMT1; water, neutralizing ions (Cl^−^) and 150 mM NaCl using CHARMM-GUI^37–40^ with default options. The protein chain N-terminus was capped by acetylation. Simulations were performed using the CHARMM36 force-field^41, 42^ in an NPγT ensemble at zero surface tension, 303.15 K temperature and 2 fs step size using NAMD 2.9^43^ on UBELIX (http://www.id.unibe.ch/hpc), the HPC cluster at the University of Bern. For analysis, STRIDE^24^ as implemented in VMD 1.9.2^44^, and MDAnalysis^45, 46^ were used, along with custom-written scripts. Figures were prepared using PyMOL 1.8.2.1^47^. For backbone dihedral angle analysis, the sum *ψ*_*i*_ + *ϕ*_*i*+1_ of the *ψ* backbone dihedral angle of residue *i* and the *ϕ*_*i*+1_ of residue *i* + *1* was calculated for each residue. For an ideal α-helix, this value is around ≈−105°.

### Human DMT1 model generation

Sequence alignment between *S*. *capitis* DMT (ScaDMT) and human DMT1 (UniProt ID: P49281–3, 1A-IRE (+) isoform) were generated using the PSI-COFFEE^48, 49^ service at Vital-IT (http://tcoffee.vital-it.ch/apps/tcoffee/do:psicoffee), with the following modifications: (i) K354-K376 in hDMT1 was modeled without a template; (ii) D377-A382 in hDMT1 was aligned with G292-L297 in ScaDMT; (iii) Y522 in hDMT1 was aligned with Y439 in ScaDMT. Residues 111–522 of hDMT1 were modeled using MODELLER 9.14^50–53^. Secondary structure restraints for α-helix on residues 357–363 were introduced based on consensus predictions by JPred4^54^, Jufo9D^55^ and PSIPRED 3.3^56, 57^ predictions. The sequence identity between ScaDMT and hDMT1 was 35.3%, reinforcing the feasibility of model construction. The best scoring model according to MODELLER objective function was chosen for subsequent studies. For MD simulations, the human DMT1 model was embedded in POPC (palmitoyl-oleoyl-phosphatidyl-choline) bilayer using CHARMM-GUI as described for ScaDMT. During the simulations, the RMSD (root mean square deviation) of C_α_ atoms from the initial model coordinates did not exceed 4 Å excluding the larger extracellular loop (residues 352–378), indicating stability of the constructed model.

## Materials

All chemicals and reagents were purchased from Sigma-Aldrich except where specified.

### Site-directed mutagenesis of human DMT1

hDMT1 mutants were generated by PCR amplification of WT hDMT1 using primers (Table 1) designed to introduce single amino-acid substitutions at positions E193, M294 and N472. For the experiments using transiently transfected human cells our previously published pIRES2 (DsRed)-hDMT1 (isoform 1A-IRE (+)) construct^36^ was used as DNA template for PCR, while for the experiments using microinjected *X*. *laevis* oocytes, the WT hDMT1 (isoform 1A-IRE (+)) was subcloned into the Pol1 vector^58^ and used as template for PCR. PCR products were digested with *Dpn I* (New England Biolabs (NEB)), which cleaves at methylated sites, allowing to digest specifically the cDNA templates but not the PCR products. XL1-Blue MRF super competent cells (Agilent technologies) were transformed with the cDNA encoding the mutants by heat-shock method and selected on LB-agar plates containing 100 µg/mL ampicillin (pIRES DsRed constructs) or 30 µg/mL kanamycin (Pol1 constructs). Mutations at desired positions were verified by DNA sequencing of the final constructs (Mycrosinth AG, Balgach, Switzerland) using specific primers (Table 1).

**Table 1:** Oligonucleotides used for site-directed mutagenesis or final product sequencing.

Mutagenesis primers	Oligonucleotide sequence
E193D	5′-GGC TCA GAC ATG CAA **GA** **C** GTC ATT GGC TCA GCC-3′
E193A	5′-GGC TCA GAC ATG CAA **G** **C** **A** GTC ATT GGC TCA GCC-3′
M294C	5′-GTG GGA GCT GTC ATC **TGC** CCA CAC AAC ATG TAC-3′
M294Q	5′-GTG GGA GCT GTC ATC **CA** **G** CCA CAC AAC ATG TAC-3′
N472Q	5′-G ATG AAT GAC TTT CTG **C** **A** **G** GTT CTA CAG AGC TTA C-3′
N472A	5′-G ATG AAT GAC TTT CTG **C** **A** **G** GTT CTA CAG AGC TTA C-3′
**Sequencing primers**	**Oligonucleotide sequence**
IRES-for	5′-TAGGCGTGTACGGTGGG-3′
IRES-rev	3′-TATAGACAAACGCACACCG-5′
M13-for	5′-TGTAAAACGACGGCCAG-3′
M13-rev	3′-CAGGAAACAGCTATGAC-5′

Mutated triplet is shown in bold, while the substituted amino acids from the Wt hDMT1 cDNA sequence are underlined. IRES primers were used to sequence pIRES2-DsRed clones, while M13 primers were used to sequence Pol1 clones.

### Cell culture and transient transfection of HEK293 cells

HEK293 cells, obtained from American Type Culture Collection (ATCC, Manassas, VA), were plated on poly-D-lysine coated 6-well or 96-well plates and maintained in Dulbecco’s modified Eagle’s medium (DMEM) (Invitrogen) supplemented with 10% fetal bovine serum (FBS) and 100 units/ml penicillin/streptomycin mixtures (Invitrogen). Cells were cultured in an incubator at 37 °C in a humidified atmosphere of 95% air and 5% CO_2_. Next day, cells were transfected with the desired cDNA and Lipofectamine 2000 (Invitrogen), as described in the manufacturer’s protocol. Functional studies and expression level determinations were performed 24 hours after transfection.

### Expression of wild-type and mutant human DMT1 in *X. laevis* oocytes

Pol1 vector containing the desired hDMT1 variant under the T7 promoter was linearized with Nhe I (NEB), and the cRNA was synthesized *in vitro* using the mMESSAGE mMACHINE T7 kit (Ambion). Defolliculated stage V-VI oocytes were micro-injected with ≈20 ng of each cRNA under study, and maintained in modified Barth’s medium (88 mM NaCl, 1 mM KCl, 2.4 mM NaHCO_3_, 0.82 mM MgSO_4_, 0.66 mM NaNO_3_, 0.75 mM CaCl_2_, 10 mM HEPES) supplemented with antibiotics. Functional studies were performed 3–5 days after micro-injection.

### Cell surface biotinylation and immunoblotting

These experiments were conducted as previously described^59^. Briefly, HEK293 cells were seeded in 6-well plates and transfected as above mentioned. After 24 hours, the cells were rinsed with PBS and incubated with 1.5 mg/mL sulfo-NHS-SS-biotin for 1 hour at 4 °C. After the labelling of the surface proteins with biotin, cells were washed with quenching buffer (standard phosphate buffers saline (PBS) buffer supplemented with 1 mM MgCl_2_, 0.1 mM CaCl_2_, and 100 mM glycine) and then rinsed once with PBS. Next, cells were lysed in radioimmunoprecipitation assay buffer (RIPA) (150 mM NaCl, 5 mM EDTA, 1% Triton X-100, 0.5% deoxycholate, 0.1% SDS, 50 mM Tris-HCl, pH 7.4) containing fresh protease inhibitor cocktail (Roche), and lysates were cleared by centrifugation. Cell lysates of equivalent amounts of protein were equilibrated overnight with streptavidin-agarose beads at 4 °C. Next day, beads were washed and recovered by centrifugation. Biotinylated protein was then released by heating to 95 °C with 2X Laemmli buffer, resolved on SDS-polyacrylamide gels, and transferred onto Immobilion-P membrane blots (Millipore). After sequential incubation of the blots with primary and secondary antibodies, proteins were revealed by enhanced chemiluminescence method (ECL). Primary antibodies were obtained from the following sources: mouse monoclonal human DMT1 (1:4000 dilution) (Abnova, Luzern, Switzerland) and rabbit polyclonal anti-actin (1:1000 dilution) (Santa Cruz Biotechnology). Secondary antibodies were obtained from the following sources: HRP-conjugated goat anti-mouse IgG (1:4000) (Bio-Rad) and goat anti-rabbit IgG (1:20000) (Promega). As a loading control, all biotinylated proteins were visualized with Avidin-HRP conjugate (Bio-Rad). To establish the relative expression levels of the different proteins, densitometry determination was performed using the National Institutes of Health software, ImageJ^60^.

### Radiotracer iron uptake

HEK293 cells were seeded in clear bottom, white-well, poly-D-lysine coated 96-well plates and transfected as described above. After 24 hours, the growth medium was removed and the cells were washed 3 times with uptake buffer (140 mM NaCl, 2.5 mM KCl, 1 mM CaCl_2_, 1 mM MgCl_2_, 1.2 mM K_2_HPO_4_, 100 mM glucose, 5 mM HEPES, 5 mM MES, pH 7.4). To measure the Fe^2+^ uptake, 100 µl of uptake buffer (pH 5.5) supplemented with the desired concentration of non-radioactive Fe^2+^, 1 mM ascorbic acid and 0.5 µCi/mL radioactive ^55^Fe^2+^ iron (American Radiolabeled Chemicals, St. Louis, MO, USA) was added into each well. The assay was performed at room temperature over 15 minutes, and the uptake was terminated by washing the plates 3 times with ice cold uptake buffer using an ELx405 microplate washer (BioTek instruments, Luzern, Switzerland). Then, 100 µL of Microscint 20 (PerkinElmer) was added to each well and incubated during 1 hour at room temperature under constant agitation. Radioactive ^55^Fe^2+^ iron uptake was determined using a TopCount Microplate Scintillation and Luminescence Counter (PerkinElmer). Counts per minute (*cpm*) were determined for each well and then transformed into influx rates using the following equation.

1 $$Influx\,rate=\frac{count/well\,(cpm)\times [substrate]\,(pM)}{initial\,total\,counts\,(cpm/L)\times uptake\,time\,(min)}$$

To obtain kinetic parameters for Fe^2+^ and H^+^ saturation kinetics, a Michaelis-Menten equation was fit to the calculated influx rates.

Radiotracer uptake in *X*. *laevis* oocytes was measured over 10 min, with up to 15 micro-injected oocytes per condition in 300 µL transport medium (100 mM NaCl, 2 mM KCl, 1 mM CaCl_2_∙2 H_2_O, 1 mM MgCl∙6 H_2_O, 5 mM HEPES and 5 m MES, pH 5.5) containing the indicated concentration of non-radioactive Fe^2+^, 1 mM ascorbic acid and 0.5 µCi/mL ^55^Fe^2+^ iron. Once the incubation time had elapsed, oocytes were rinsed 3 times with ice-cold transport medium (pH 5.5) containing 250 µM non-radioactive Fe^2+^ and 1 mM ascorbic acid. Then, oocytes were solubilized with SDS 10% and the content of ^55^Fe^2+^ was determined by liquid scintillation counting. To determine background signal of the measurements, uptake by non-injected oocytes was determined for all the conditions tested. Influx rates were calculated as mentioned above.

### Cadmium influx measured by real-time fluorescence imaging

HEK293 cells were seeded in clear bottom, black-well, poly-D-lysine coated 96-well plates and transfected as described above. 24 hours after transfection, growth media was removed and cells were loaded with Calcium 5 fluorescent dye (Molecular Devices) in 100 µL of uptake buffer (117 mM NaCl, 4.8 mM KCl, 1 mM MgCl, 10 mM glucose, 5 mM HEPES, 5 mM MES, pH 7.4) at 37 °C for 1 hour. Measurements were performed at 37 °C using the FLIPR Tetra fluorescence microplate reader (Molecular Devices) as previously reported^36^. Cells were excited using a 470 to 495 nm LED module, and the emitted fluorescence signal was filtered with a 515 to 575 emission filter. Fluorescence signals were analyzed using the FLIPR Tetra software (ScreenWorks 3.1.2.002). A stable baseline was established for 50 seconds, then, 50 µL of uptake buffer (pH 6.5) containing 2 µM Cd^2+^ was added and changes in fluorescence intensity were measured for 600 seconds. To measure the hDMT1 activity, the increase of the fluorescence intensity in response to cadmium was quantified by calculating the area under the curve.

### Electrophysiology methods

The two-electrode voltage-clamp method was used to measure currents associated to the functional activity of wild type or mutant DMT1 expressed in *X*. *laevis* oocytes as previously described^10, 28^. Briefly, oocytes were placed in the recording chamber, which was continuously perfused with uptake buffer (117 mM NaCl, 4.8 mM KCl, 1 mM MgCl, 10 mM glucose, 5 mM HEPES, 5 mM MES, pH 7.4), and oocytes were impaled with two glass micro-electrodes filled with 3 mM KCl (resistance between 0.2 and 2 mΩ). Voltage-clamp experiments were performed according to two different protocols: (i) Oocytes were clamped at the holding potential (*V*_*h*_ = −50 mV), and step-changes in membrane potential (*V*_*m*_) were applied from +50 to −150 mV (in increments of 20 mV) each for a duration of 200 ms, before and after the addition of Fe^2+^. Steady-state currents were obtained by averaging the points over the middle point of the trace (10 ms) at each *V*_*m*_ step. Substrate-induced currents were determined as the difference between the steady-state currents measured in the absence and presence of Fe^2+^. (ii) Pre-steady state currents were recorded in the absence of Fe^2+^ using a modified version of protocol (i), in which the voltage steps were applied from + 90 to −110 mV. In both cases, currents were measured with an OC-725 amplifier (Warner Instruments), low-pass filtered at 500 Hz, digitized at 5 kHz with a Digidata 1440 data acquisition system and captured using pClamp 10 software (Axon Instruments).

The following 4-parameter Hill function was fit to substrate-induced current data, for which *I* is the evoked current, *I*_*max*_ is the derived maximum current, *S* is the concentration of substrate (H^+^ or Fe^2+^), *K* ^*S*^_0.5_ is the substrate concentration at which current was half-maximal, *n*_*H*_ is the Hill coefficient for *S*, and *i* ^*U*^ describes the Fe^2+^-evoked current in the absence of H^+^.

2 $$I={i}^{U}+\frac{{I}_{max}\,{S}^{{n}_{H}}}{{({K}_{0.5}^{S})}^{{n}_{H}}+{S}^{{n}_{H}}}$$

Following step-changes of the above described protocol (ii), pre-steady state currents were obtained. These currents had to be isolated from capacitive transient currents and steady-state currents by the previously described fitted method^10, 28, 61^. Obtained pre-steady state currents were then integrated with time to determine charge movements (*Q*) and these were used to fit the following Boltzmann function for which maximal charge *Q*_*max*_ = *Q*_*dep*_ − *Q*_*hyp*_ (*Q*_*dep*_ and *Q*_*hyp*_ represent the charge at depolarizing and hyperpolarizing limits, respectively), *V*_0.5_ is the *V*_*m*_ at the midpoint of charge transfer, *z* is the apparent valence of the movable charge, and *F*, *R* and *T* have their standard thermodynamic meanings.

3 $$\frac{Q-{Q}_{hyp}}{{Q}_{max}}=\frac{1}{1+\exp [z({V}_{m}-{V}_{0.5})F/RT]}$$

### Statistics

Except for representative experiments, results are expressed as mean values ± standard deviation (SD). Difference between independent groups were assessed by unpaired Student’s test or Mann-Whitney U test, depending on the fit of the dependent variables to the normal distribution assessed by Kolmogorov-Smirnov and Shapiro-Wilk tests (for experiments with less than 50 samples). All statistical tests were performed using the IBM SPSS statistics 20 software. Significance level was set at *p* < 0.05.

## Electronic supplementary material

Supplementary Information

## References

[1] Ganz T (2013). Systemic iron homeostasis. Physiol. Rev..

[2] Canonne-Hergaux F, Gruenheid S, Ponka P, Gros P (1999). Cellular and subcellular localization of the Nramp2 iron transporter in the intestinal brush border and regulation by dietary iron. Blood.

[3] Fleming MD (1998). Nramp2 is mutated in the anemic Belgrade (b) rat: evidence of a role for Nramp2 in endosomal iron transport. Proc. Natl. Acad. Sci. USA.

[4] Fleming MD (1997). Microcytic anaemia mice have a mutation in Nramp2, a candidate iron transporter gene. Nat. Genet..

[5] Iolascon A (2008). Natural history of recessive inheritance of DMT1 mutations. J. Pediatr..

[6] Blanco E, Kannengiesser C, Grandchamp B, Tasso M, Beaumont C (2009). Not all DMT1 mutations lead to iron overload. Blood Cells. Mol. Dis..

[7] Gunshin H (1997). Cloning and characterization of a mammalian proton-coupled metal-ion transporter. Nature.

[8] Illing AC, Shawki A, Cunningham CL, Mackenzie B (2012). Substrate profile and metal-ion selectivity of human divalent metal-ion transporter-1. J. Biol. Chem..

[9] Chen XZ (1999). Yeast SMF1 mediates H(+)-coupled iron uptake with concomitant uncoupled cation currents. J. Biol. Chem..

[10] Mackenzie B, Ujwal ML, Chang M-H, Romero MF, Hediger MA (2006). Divalent metal-ion transporter DMT1 mediates both H+ -coupled Fe2+ transport and uncoupled fluxes. Pflüg. Arch. Eur. J. Physiol..

[11] Ehrnstorfer IA, Geertsma ER, Pardon E, Steyaert J, Dutzler R (2014). Crystal structure of a SLC11 (NRAMP) transporter reveals the basis for transition-metal ion transport. Nat. Struct. Mol. Biol..

[12] Bozzi AT (2016). Crystal Structure and Conformational Change Mechanism of a Bacterial Nramp-Family Divalent Metal Transporter. Struct. Lond. Engl. 1993.

[13] Ehrnstorfer IA, Manatschal C, Arnold FM, Laederach J, Dutzler R (2017). Structural and mechanistic basis of proton-coupled metal ion transport in the SLC11/NRAMP family. Nat. Commun..

[14] Krishnamurthy H, Gouaux E (2012). X-ray structures of LeuT in substrate-free outward-open and apo inward-open states. Nature.

[15] Gur M, Zomot E, Cheng MH, Bahar I (2015). Energy landscape of LeuT from molecular simulations. J. Chem. Phys..

[16] Perez C, Ziegler C (2013). Mechanistic aspects of sodium-binding sites in LeuT-like fold symporters. Biol. Chem..

[17] Shi L, Weinstein H (2010). Conformational rearrangements to the intracellular open states of the LeuT and ApcT transporters are modulated by common mechanisms. Biophys. J..

[18] Shaffer PL, Goehring A, Shankaranarayanan A, Gouaux E (2009). Structure and mechanism of a Na+ -independent amino acid transporter. Science.

[19] Schulze S, Köster S, Geldmacher U, Terwisscha van Scheltinga AC, Kühlbrandt W (2010). Structural basis of Na(+)-independent and cooperative substrate/product antiport in CaiT. Nature.

[20] Kalayil S, Schulze S, Kühlbrandt W (2013). Arginine oscillation explains Na+ independence in the substrate/product antiporter CaiT. Proc. Natl. Acad. Sci. USA.

[21] Huang Z, Tajkhorshid E (2010). Identification of the third Na+ site and the sequence of extracellular binding events in the glutamate transporter. Biophys. J..

[22] Larsson HP (2010). Evidence for a third sodium-binding site in glutamate transporters suggests an ion/substrate coupling model. Proc. Natl. Acad. Sci. USA.

[23] Zhao C, Noskov SY (2013). The Molecular Mechanism of Ion-Dependent Gating in Secondary Transporters. PLoS Comput Biol.

[24] Frishman D, Argos P (1995). Knowledge-based protein secondary structure assignment. Proteins.

[25] Shi L, Quick M, Zhao Y, Weinstein H, Javitch JA (2008). The mechanism of a neurotransmitter:sodium symporter–inward release of Na+ and substrate is triggered by substrate in a second binding site. Mol. Cell.

[26] Haemig HAH, Brooker RJ (2004). Importance of Conserved Acidic Residues in MntH, the Nramp homolog of Escherichia coli. J. Membr. Biol..

[27] Bozzi AT (2016). Conserved methionine dictates substrate preference in Nramp-family divalent metal transporters. Proc. Natl. Acad. Sci. USA.

[28] Mackenzie B, Takanaga H, Hubert N, Rolfs A, Hediger MA (2007). Functional properties of multiple isoforms of human divalent metal-ion transporter 1 (DMT1). Biochem. J..

[29] Loo DD, Hazama A, Supplisson S, Turk E, Wright EM (1993). Relaxation kinetics of the Na+/glucose cotransporter. Proc. Natl. Acad. Sci. USA.

[30] Parent L, Supplisson S, Loo DD, Wright EM (1992). Electrogenic properties of the cloned Na+/glucose cotransporter: I. Voltage-clamp studies. J. Membr. Biol..

[31] Marcoline FV, Bethel N, Guerriero CJ, Brodsky JL, Grabe M (2015). Membrane Protein Properties Revealed through Data-Rich Electrostatics Calculations. Struct. Lond. Engl. 1993.

[32] Søndergaard CR, Olsson MHM, Rostkowski M, Jensen JH (2011). Improved Treatment of Ligands and Coupling Effects in Empirical Calculation and Rationalization of pKa Values. J. Chem. Theory Comput..

[33] Olsson MHM, Søndergaard CR, Rostkowski M, Jensen JH (2011). PROPKA3: Consistent Treatment of Internal and Surface Residues in Empirical pKa Predictions. J. Chem. Theory Comput..

[34] Callenberg, K. M. *et al*. APBSmem: a graphical interface for electrostatic calculations at the membrane. *PloS One* **5** (2010). 10.1371/journal.pone.0012722 PMC2947494 20949122

[35] Lomize MA, Lomize AL, Pogozheva ID, Mosberg HI (2006). OPM: orientations of proteins in membranes database. Bioinforma. Oxf. Engl..

[36] Montalbetti N, Simonin A, Dalghi MG, Kovacs G, Hediger MA (2014). Development and Validation of a Fast and Homogeneous Cell-Based Fluorescence Screening Assay for Divalent Metal Transporter 1 (DMT1/SLC11A2) Using the FLIPR Tetra. J. Biomol. Screen..

[37] Jo S, Kim T, Iyer VG, Im W (2008). CHARMM-GUI: A web-based graphical user interface for CHARMM. J. Comput. Chem..

[38] Lee J (2016). CHARMM-GUI Input Generator for NAMD, GROMACS, AMBER, OpenMM, and CHARMM/OpenMM Simulations Using the CHARMM36 Additive Force Field. J. Chem. Theory Comput..

[39] Jo S (2014). CHARMM-GUI PDB manipulator for advanced modeling and simulations of proteins containing nonstandard residues. Adv. Protein Chem. Struct. Biol..

[40] Wu EL (2014). CHARMM-GUI Membrane Builder toward realistic biological membrane simulations. J. Comput. Chem..

[41] Klauda JB (2010). Update of the CHARMM all-atom additive force field for lipids: validation on six lipid types. J. Phys. Chem. B.

[42] Best, R. B. *et al*. Optimization of the additive CHARMM all-atom protein force field targeting improved sampling of the backbone φ, ψ and side-chain χ(1) and χ(2) dihedral angles. *J*. *Chem*. *Theory Comput*. **8**, 3257–3273 (2012). 10.1021/ct300400x PMC3549273 23341755

[43] Phillips JC (2005). Scalable molecular dynamics with NAMD. J. Comput. Chem..

[44] Humphrey, W., Dalke, A. & Schulten, K. VMD: visual molecular dynamics. *J*. *Mol*. *Graph*. **14**, 33–38, 27–28 (1996). 10.1016/0263-7855(96)00018-5 8744570

[45] Michaud-Agrawal N, Denning EJ, Woolf TB, Beckstein O (2011). MDAnalysis: a toolkit for the analysis of molecular dynamics simulations. J. Comput. Chem..

[46] Gowers, R. J. *et al*. MDAnalysis: A Python Package for the Rapid Analysis of Molecular Dynamics Simulations. in *Proceedings of the 15th Python in Science Conference* (eds Benthall, S. & Rostrup, S.) 98–105 (2016).

[47] Schrödinger, L. L. C. The PyMOL Molecular Graphics System, Version 1.8. (2015).

[48] Notredame C, Higgins DG, Heringa J (2000). T-Coffee: A novel method for fast and accurate multiple sequence alignment. J. Mol. Biol..

[49] Kemena C, Notredame C (2009). Upcoming challenges for multiple sequence alignment methods in the high-throughput era. Bioinforma. Oxf. Engl..

[50] Sali A, Blundell TL (1993). Comparative protein modelling by satisfaction of spatial restraints. J. Mol. Biol..

[51] Fiser A, Do RK, Sali A (2000). Modeling of loops in protein structures. Protein Sci. Publ. Protein Soc..

[52] Martí-Renom MA (2000). Comparative protein structure modeling of genes and genomes. Annu. Rev. Biophys. Biomol. Struct..

[53] Webb B, Sali A (2014). Comparative Protein Structure Modeling Using MODELLER. Curr. Protoc. Bioinforma. Ed. Board Andreas Baxevanis Al.

[54] Drozdetskiy A, Cole C, Procter J, Barton GJ (2015). JPred4: a protein secondary structure prediction server. Nucleic Acids Res..

[55] Leman JK, Mueller R, Karakas M, Woetzel N, Meiler J (2013). Simultaneous prediction of protein secondary structure and transmembrane spans. Proteins.

[56] Jones DT (1999). Protein secondary structure prediction based on position-specific scoring matrices. J. Mol. Biol..

[57] Buchan DWA, Minneci F, Nugent TCO, Bryson K, Jones DT (2013). Scalable web services for the PSIPRED Protein Analysis Workbench. Nucleic Acids Res..

[58] Bergeron MJ, Bürzle M, Kovacs G, Simonin A, Hediger MA (2011). Synthesis, maturation, and trafficking of human Na+ -dicarboxylate cotransporter NaDC1 requires the chaperone activity of cyclophilin B. J. Biol. Chem..

[59] Simonin A, Fuster D (2010). Nedd4-1 and beta-arrestin-1 are key regulators of Na+/H+ exchanger 1 ubiquitylation, endocytosis, and function. J. Biol. Chem..

[60] Wayne S. R. *ImageJ*. (National Institutes of Health, 1997).

[61] Hazama A, Loo DD, Wright EM (1997). Presteady-state currents of the rabbit Na+/glucose cotransporter (SGLT1). J. Membr. Biol..

[62] Kozma D, Simon I, Tusnády GE (2013). PDBTM: Protein Data Bank of transmembrane proteins after 8 years. Nucleic Acids Res..

