# Supplementary material for: A novel proton transfer mechanism in the SLC11 family of divalent metal ion transporters
Source: Sci Rep. 2017 Jul 28;7:6194. doi: 10.1038/s41598-017-06446-y (PMC5533754; doi:10.1038/s41598-017-06446-y)

Supplementary information

A novel proton transfer mechanism in the SLC11 family of divalent metal ion transporters

Jonai Pujol-Giménez[1], Matthias A. Hediger[1] and Gergely Gyimesi[1]

[1]Institute of Biochemistry and Molecular Medicine and National Center of Competence in Research, NCCR TransCure, University of Bern, Switzerland

Supplementary Figure S1. Comparison with leucine transporter LeuT. **(A)** Comparison of the vicinity of the bound Na2 ion in an outward-facing state of LeuT (right panel) and its analogous location in ScaDMT (left panel), indicating the E127 residue. TMH 5 was hidden for clarity. **(B)** Helicity changes upon protonation of E112 in two different states of LeuT were quantitated by calculating the time average of the sum of dihedral angles $\psi_i + \phi_{i+1}$ along the protein chain for five independent molecular dynamics trajectories. For an ideal α-helix, this value is ≈−105°.

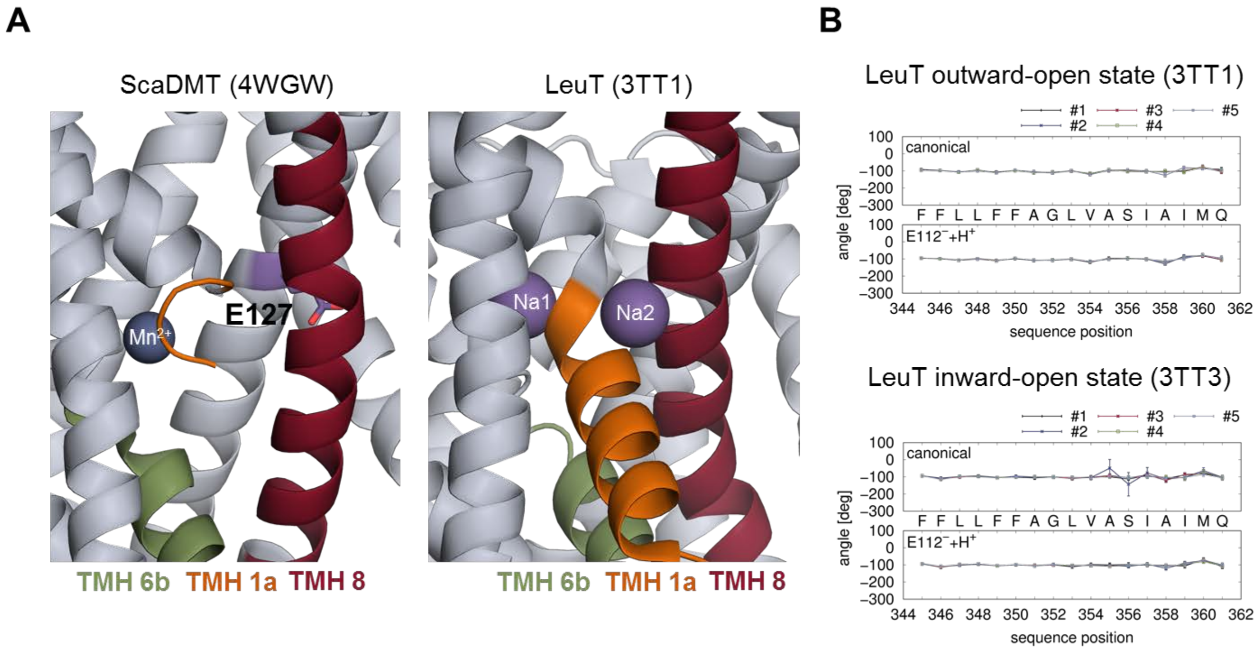

Supplementary Figure S2. Secondary structure in doubly protonated systems. **(A)** Time average of helicity of each residue as observed in simulations of ScaDMT in various doubly protonated states, quantified using STRIDE. The indicated transmembrane regions were taken from the PDBTM database. **(B)** Regions near TMH 4-5 (left), TMH 6 (center) and TMH 8 (right) are shown. **(C)** Helicity changes in each MD trajectory were also quantitated by calculating the time average of the sum of dihedral angles $\psi_i + \phi_{i+1}$ along the protein chain. For an ideal α-helix, this value is ≈−105°. Large values around 200° correspond to local peptide bond flips.

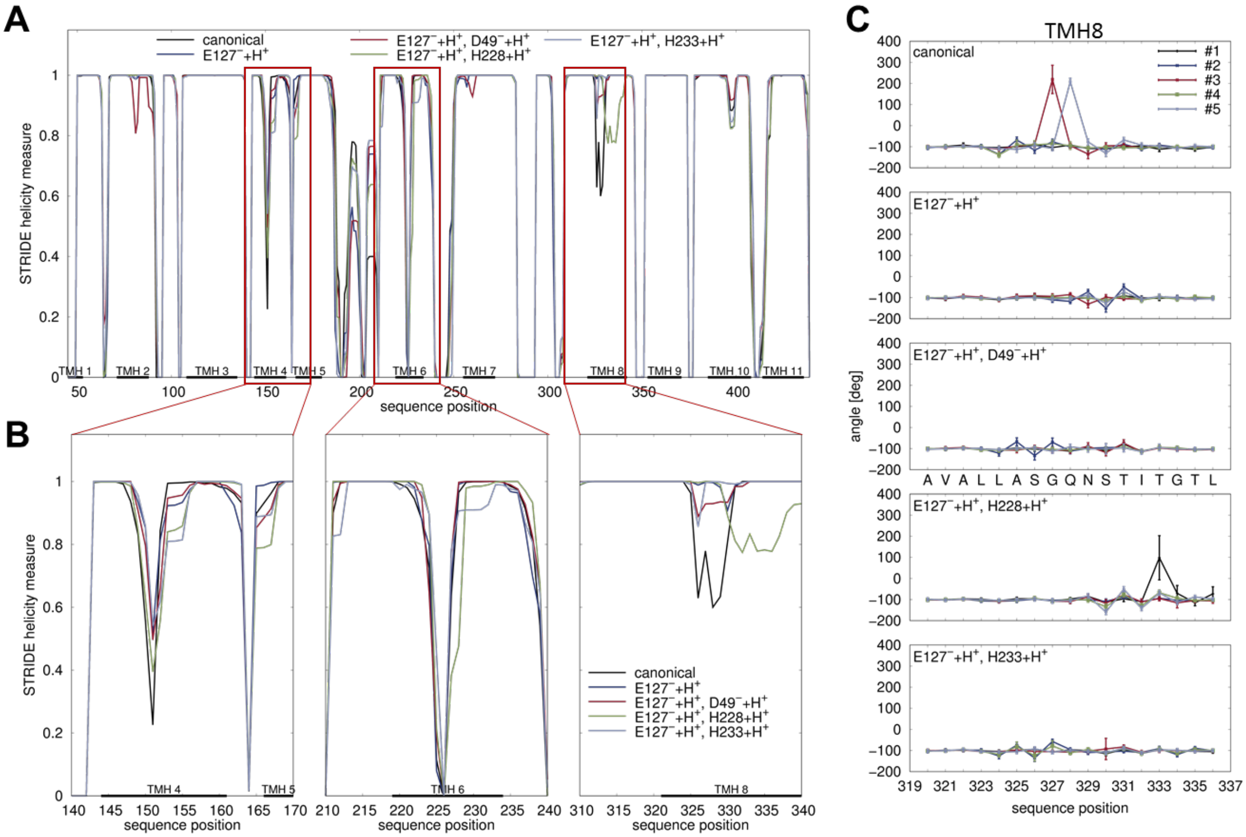

Supplementary Figure S3. Model of human DMT1. **(A)** The model of human DMT1 built based on the ScaDMT structure is shown. **(B)** Side-chain pK_a values of residues analyzed in ScaDMT also show similar values in hDMT1. (C) TMH 8 in hDMT1 partially unwinds in 2 out of 5 “canonical” simulation and can be stabilized by protonating E193 (corresponding to E127 in ScaDMT), similarly to ScaDMT.

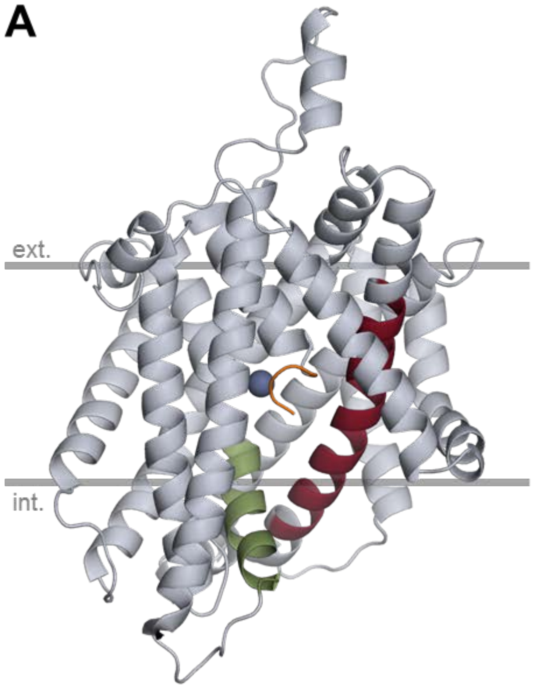

B

ScaDMT Residue		hDMT1 Residue		hDMT1 pK_a	
				+ Mn^{2+}	− Mn^{2+}
ASP	49	ASP	115	2.28	6.50
GLU	127	GLU	193	9.40	9.52
HIS	228	HIS	296	3.53	4.28
HIS	233	HIS	301	1.44	1.54

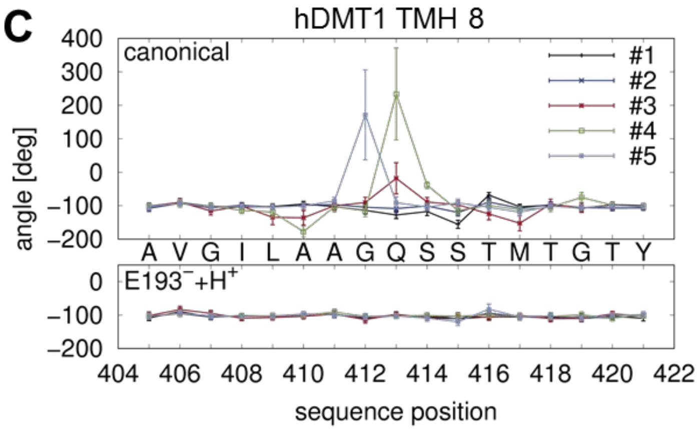

Supplementary Figure S4. H^+-leak saturation kinetics of hDMT1 and E193 expressed in* X. laevis *oocytes. **(A)** H^+-leak-voltage relationship (V_h = −50 mV; pH 5.5). H^+-leak currents were measured as the shift in the baseline current when switching from pH 7.7 to pH 5.5. **(B)** Representative experiment of H^+-leak currents (V_h = −50 mV; V_t = −70 mV; pH 5.5) as a function of [H^+]. Kinetic parameters of H^+-leak were calculated by fitting a 4-parameter sigmoidal equation (connecting lines) to the data and are summarized below the graph. **(C)** H^+-leak $K_{0.5}$ as a function of membrane potential (V_m), each $K_{0.5}$ value corresponds to the mean ± S.D. calculated from 5–9 oocytes obtained from 3 different batches.

A B

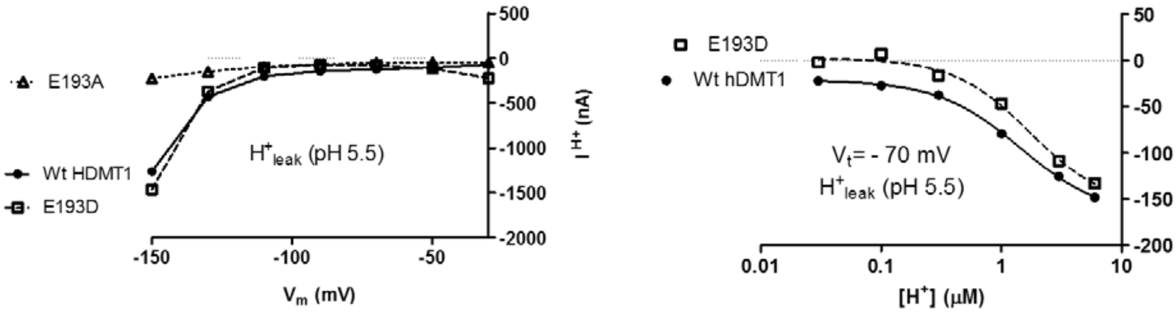

Isoform	I_{max} (nA)	EC_{50} (μM)	N_H	r^2
WT hDMT1	−168 ± 6.6	1.45 ± 0.03	1.2 ± 0.1	0.99
E193D	−159 ± 32	1.74 ± 0.16	1.3 ± 0.5	0.99

C

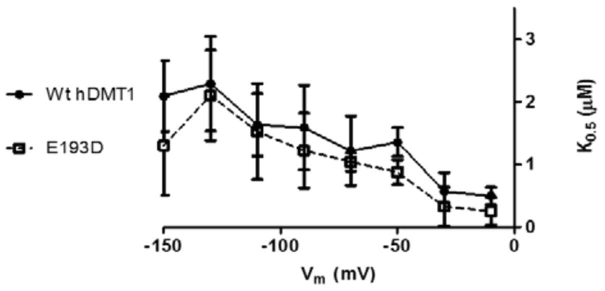

Supplementary Figure S5. TMH 8 helicity and accessible rotameric states of E127 in ScaDMT with artificially neutralized E127 side-chain. **(A)** Time average of helicity of residues in TMH 8 as quantified by STRIDE (left panel) and backbone dihedral analysis (right panel), in various simulations of ScaDMT. “E127 neutral” refers to simulations with artificially neutralized and deprotonated E127 side-chain. The “canonical” and “$E127^{-}+H^{+}$” graphs are identical to those in Figure 2B and are shown for comparison. **(B)** Accessible states of the E127 side-chain are shown by mapping the individual simulation trajectories into the space of the χ_1-χ_2 dihedral angles (see Figure 9). Trajectory of the neutralized E127 systems are shown on the middle panel. Left and right panels showing the “canonical” and “$E127^{-}+H^{+}$” simulation systems are identical to those in Figure 9C and D, respectively, and are shown for comparison.

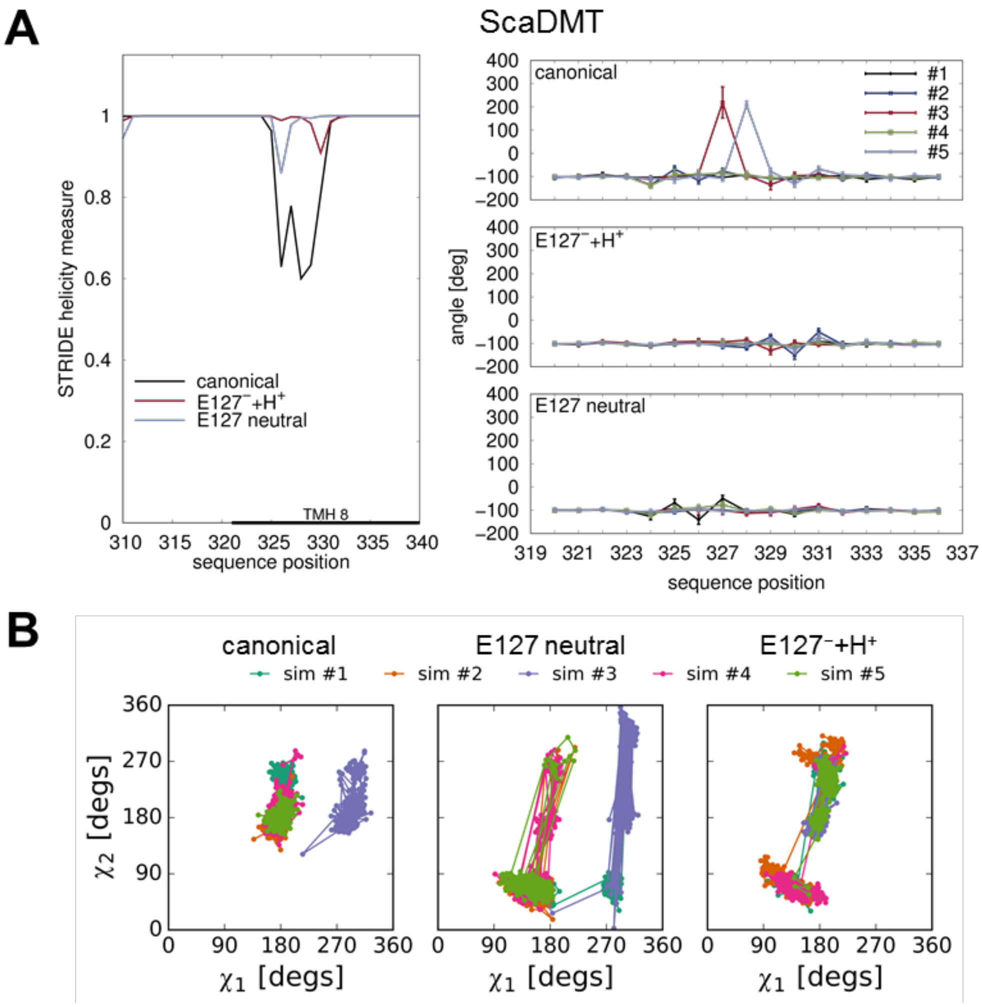

Supplementary Figure S6. Stability and side-chain pK_a of E127D and E193D mutants. **(A)** Backbone helicity analysis of wild-type and variants of E127 (ScaDMT, left) and E193 (hDMT1, right) in the TMH 8 region. Top two panels for ScaDMT and hDMT1 reproduced from Figures 2B and S3C, respectively, and are shown for comparison. Results for the protonated D127 and D193 side-chain are shown on the bottom panels. **(B)** Cluster centroid conformations of side-chains D127 (ScaDMT, left) and D193 (hDMT1, right) are shown with regard to angle χ_1, along with calculated pK_a values for the depicted conformations.

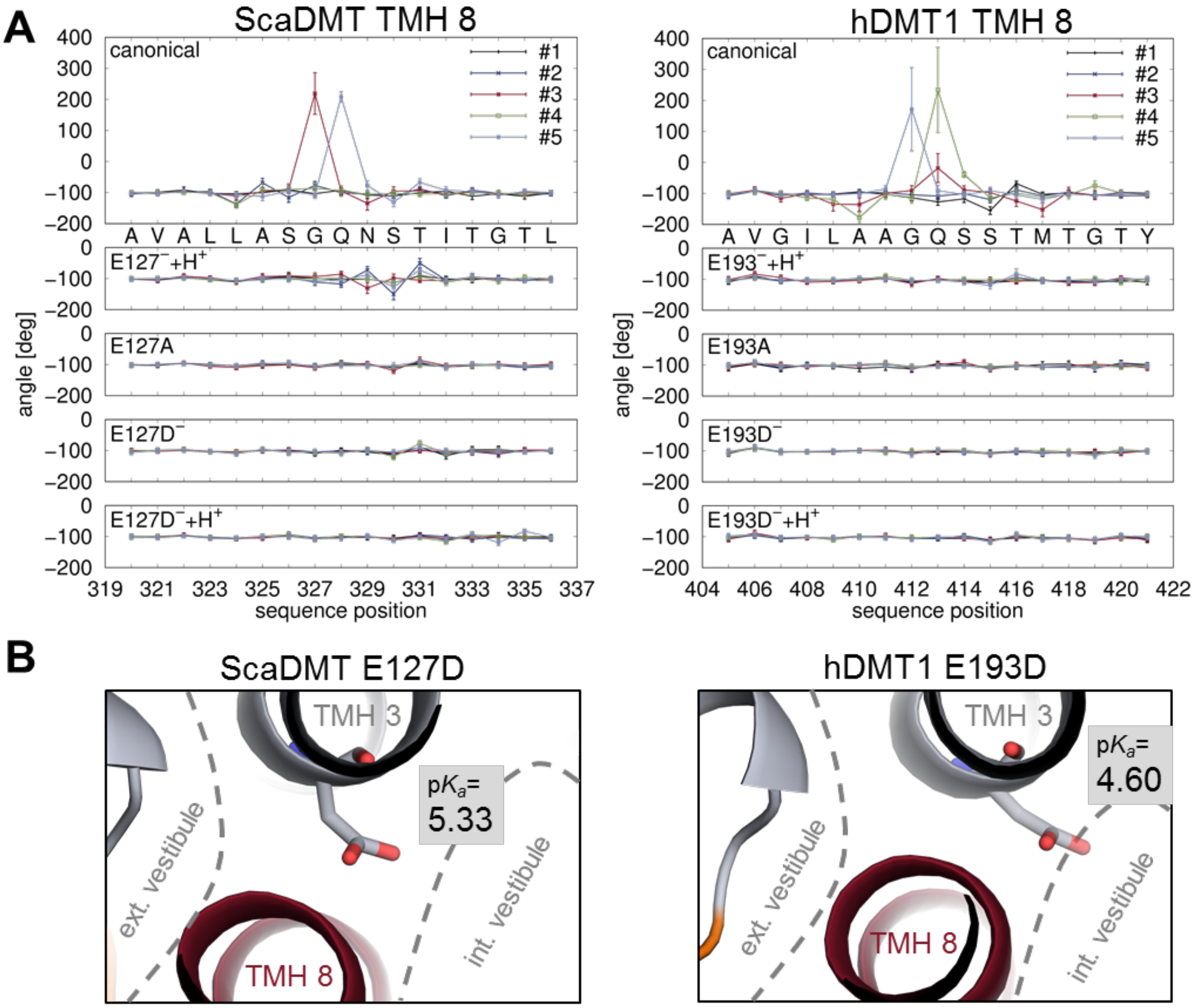

Supplement: Supplementary file 1 — Supplementary Information [file 41598_2017_6446_MOESM1_ESM.pdf]
